# Selective Inhibition Mediates the Sequential Recruitment of Motor Pools

**DOI:** 10.1016/j.neuron.2016.07.041

**Published:** 2016-08-17

**Authors:** Maarten F. Zwart, Stefan R. Pulver, James W. Truman, Akira Fushiki, Richard D. Fetter, Albert Cardona, Matthias Landgraf

(Neuron *91*, 615–628; August 3, 2016)

We regret and hereby correct the omission of Richard D. Fetter as an author, who performed sample preparation, serial sectioning and electron microscopy imaging of the whole *Drosophila* larval central nervous system, making possible the subsequent neuron reconstructions and synapse annotations of larval premotor circuits. We also acknowledge the Fly EM Project Team technicians who assisted R.D.F in imaging the serial sections with the statement: “We thank the Fly EM Project Team for assisting R.D.F. with imaging the serial sections of the larval central nervous system with transmission electron microscopy.” This has been corrected both online and in print.

