# Supplementary material for: Selective Inhibition Mediates the Sequential Recruitment of Motor Pools
Source: Neuron. 2016 Aug 3;91(3):615–28. doi: 10.1016/j.neuron.2016.06.031 (PMC4980426; doi:10.1016/j.neuron.2016.06.031)
Supplement: Document S1. Figures S1–S6 [file mmc1.pdf]

Neuron, Volume *91*

Supplemental Information

Selective Inhibition Mediates the Sequential Recruitment of Motor Pools

Maarten F. Zwart, Stefan R. Pulver, James W. Truman, Akira Fushiki, Richard D. Fetter, Albert Cardona, and Matthias Landgraf

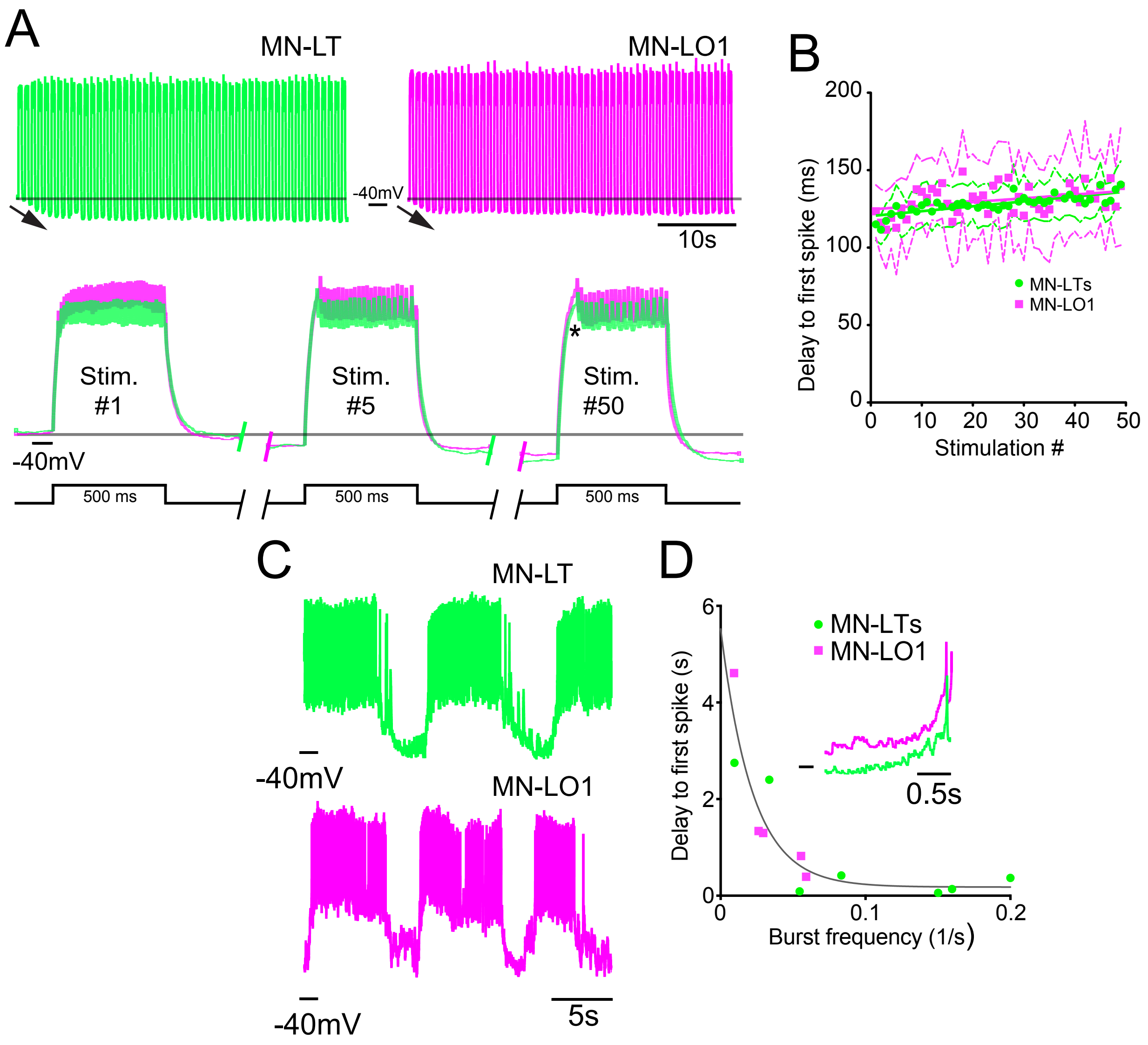

Figure S1, related to Figure 1. Motor neuron intrinsic properties do not contribute to the generation of the intrasegmental motor pattern underlying larval crawling.

(A) Top panels represent example traces of current clamp recordings of an MN-LT and MN-LO1 while repeatedly injecting 20 pA of current for 500 ms per stimulation. Horizontal line indicates resting membrane potential before experiment; arrow emphasizes downward trend after repeated stimulation. Bottom panels show overlaid traces of experiment for stimulation #1, #5 and #50. Asterisk in #50 indicates the change in the delay to first spike. (B) Quantification of delay to first spike as a function of stimulation number for MN-LT and MN-LO1. Plot shows mean delay to first spike (squares, circles) ± SEM (dashed lines). Solid lines indicate linear regression fits. There is no statistically significant difference between either the slopes (p=0.77) or intercepts (p=0.51) of the two fits. n=5 for MN-LO1, n=9 for MN-LTs. (C) Example traces of current clamp recordings of an MN-LT and MN-LO1 during fictive crawling in two different preparations. Motoneurons fire action potentials as the result of endogenous activity within the motor system. (D) Quantification of delay to first spike, as measured from the start of depolarization to the first action potential, as a function of burst frequency. Solid grey line indicates non-linear fit of the data. One curve fits both data sets best (p=0.17). Inset is expanded view of traces in (C) showing similar delay to first spike. n=5 for MN-LO1, n=7 for MNs-LT.

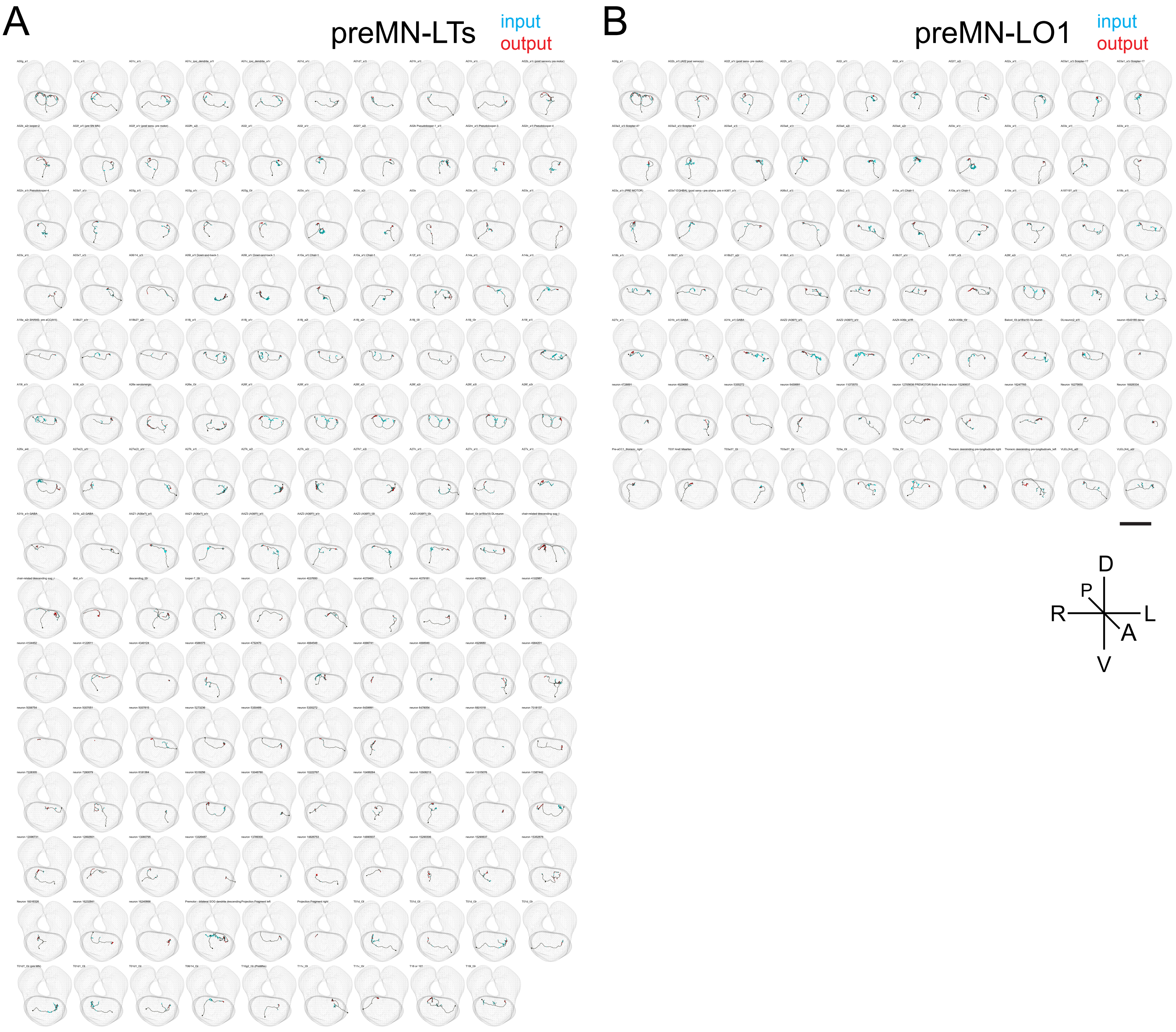

Figure S2, related to Figure 2. Functionally distinct motor neurons receive divergent input.
(A, B) Anterior views of individual reconstructions of all premotor interneurons reconstructed for this study. Scale bar indicates 50 µm.

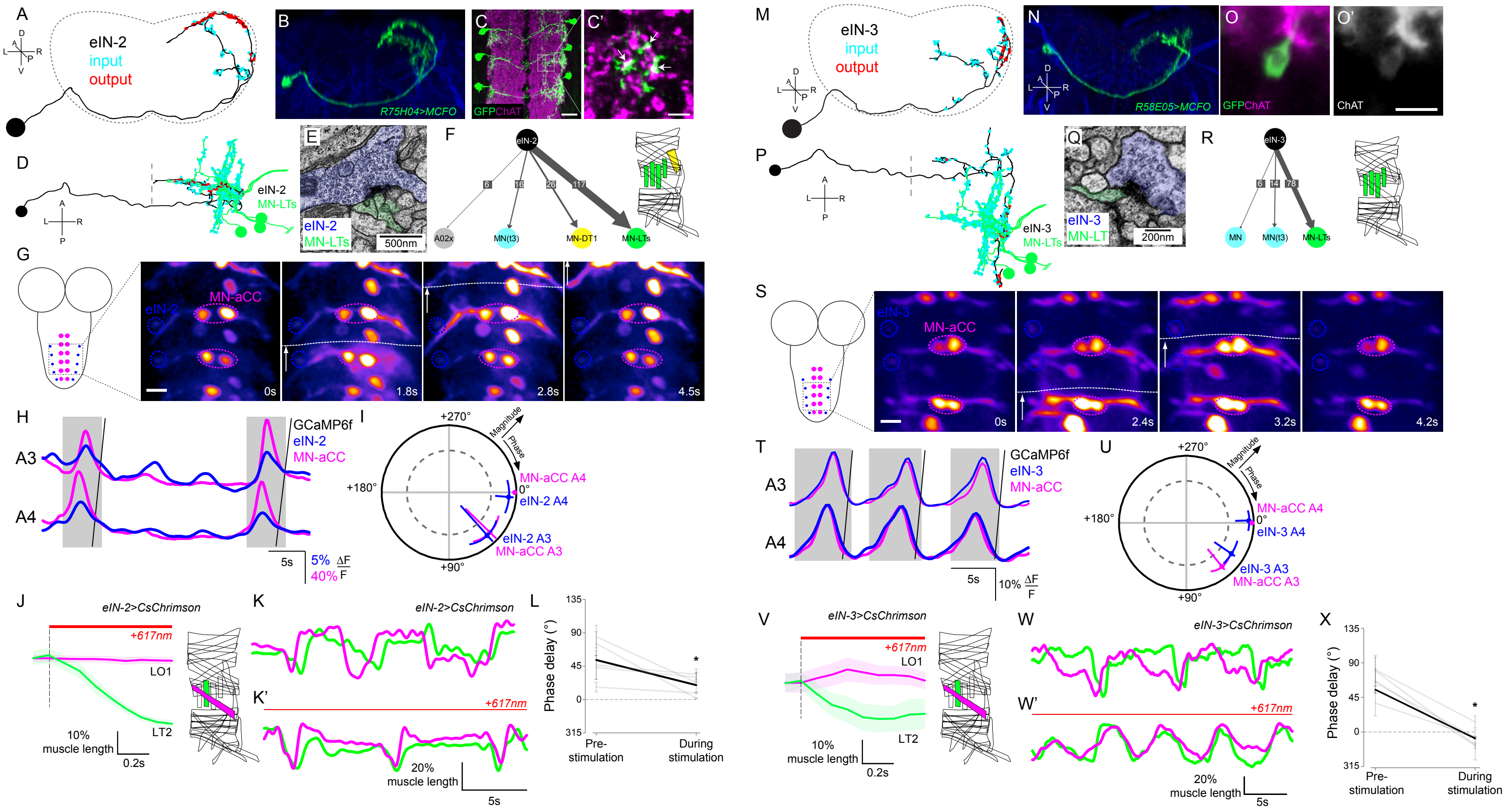

Figure S3, related to Figure 3. eIN-2 and eIN-3 innervate MN-LTs and are recruited during locomotion.
Posterior view of EM reconstruction and light microscopy data of eIN-2 (A,B) and eIN-3 (M,N). (C, O) Immunohistochemical labeling of SS02065>myrGFP (C) and SS01379>GFP (O) showing pronounced ChAT staining. (D, P) Dorsal view of an eIN-2 (D) and eIN-3 (P) innervating contralateral MN-LT1 - MN-LT4. (E, Q) Electron micrograph showing the apposition of eIN-2 and two LT motoneurons (E) and an eIN-3 with a single MN-LT (Q), with synaptic vesicles and the presynaptic density clearly visible. (F, R) Connectivity diagram of eIN-2 and eIN-3. eIN-2 also innervates the DT1 motoneuron, which innervates a muscle of similar orientation as the MNs-LT. Blue motoneuron innervates unknown muscles (segmental identity in brackets), grey node indicates interneuron. Included in this diagram are all postsynaptic neurons with a connection to eIN-2 and eIN-3 of more than 5 synapses. Muscle diagram indicates identity of known target muscles. (G-I, S-U) eIN-2 and eIN-3 show wave-like activity during fictive crawling. (G, S) Stills showing GCaMP6f activity in eIN-2 and eIN-3 and aCC motoneurons as indicated in schematic, quantified in (H) and (T). (I, U) Coherency between eIN-2 and eIN-3 and aCC motoneurons in segments A3 and A4. (J, V) Acute high-intensity optogenetic stimulation (617 nm, 1.1mW/mm^2) of eIN-2 and eIN-3 induces contraction of the transverse muscles, as measured by muscle-imaging experiments in semi-intact preparations (see Experimental Procedures). LO1 does not contract in response to optogenetic stimulation of eIN-2 or eIN-3. (K, W) Low-level chronic stimulation of eIN-2 and eIN-3 (617 nm, 0.01 mW/mm^2) causes the transverse muscles to contract earlier in the locomotor cycle. (K, W) Quantification of contraction of LO1 and LT2 muscles pre-stimulation, (K', W') during stimulation. (L, X) The phase delay between LO1 and LT2 is reduced in response to low-level chronic stimulation of eIN-2 (L) and eIN-3 (X). Grey lines indicate individual preparations, black line represents mean. Hotelling paired test, $p<0.05$ for (L) and (X). n=10 stimulations for (J) and (V), n=5 animals for all other experiments. Data are represented as mean ± 95% CI in (I) and (U), mean ± SD in (J), (L), (V), and (X). Scale bars in (C), (G), (O'), and (S) indicate 10µm, 1µm in (C').

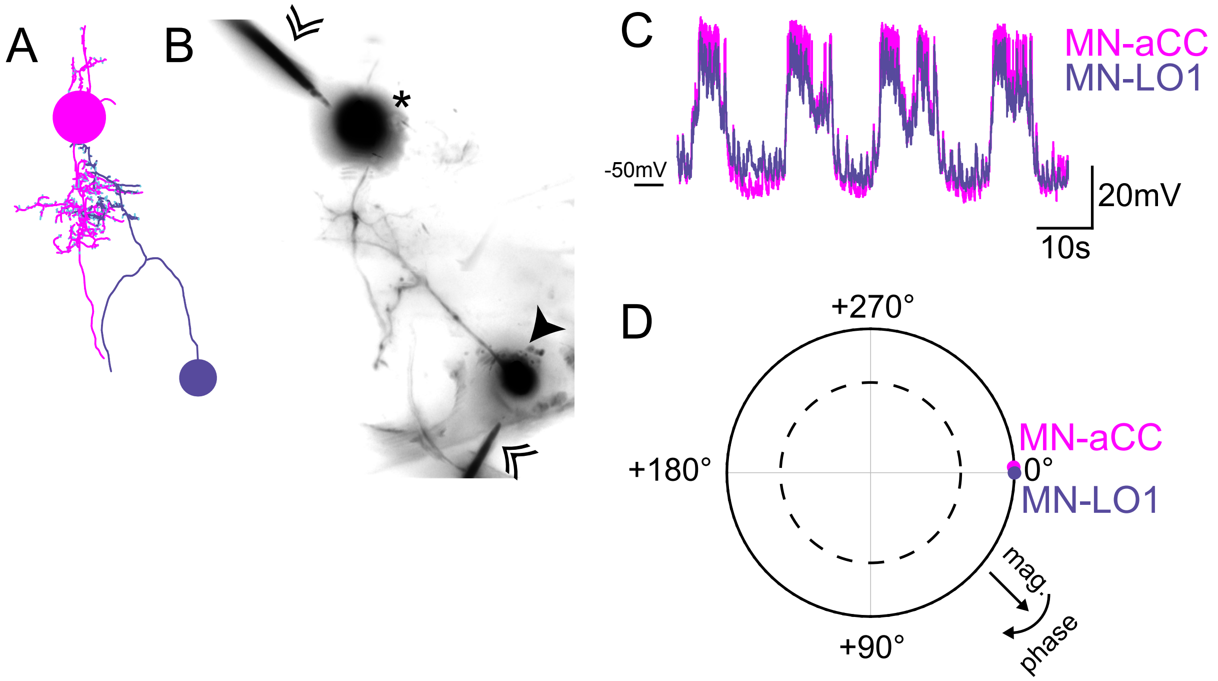

Figure S4, related to Figure 3. MN-LO1 is active in phase with MN-aCC.
(A) Dorsal view of EM reconstruction of MN-aCC (magenta) and MN-LO1 (violet) within the same segment. (B) Example recording of an MN-aCC (asterisk) and an MN-LO1 (arrowhead), showing recording electrodes (chevrons). Cells are visualized by Alexa 568 dye added to the intracellular solution. (C) Traces of simultaneous whole cell recording in current clamp of MN-aCC (magenta) and MN-LO1 (violet), showing membrane voltage fluctuations and action potentials that occur as the result of spontaneous fictive crawling. (D) Coherency between MN-aCC motoneuron and MN-LO1. MN-aCC and MN-LO1 are highly coherent with one another and are very close in phase. Data are represented as mean ± 95% CI, n=4.

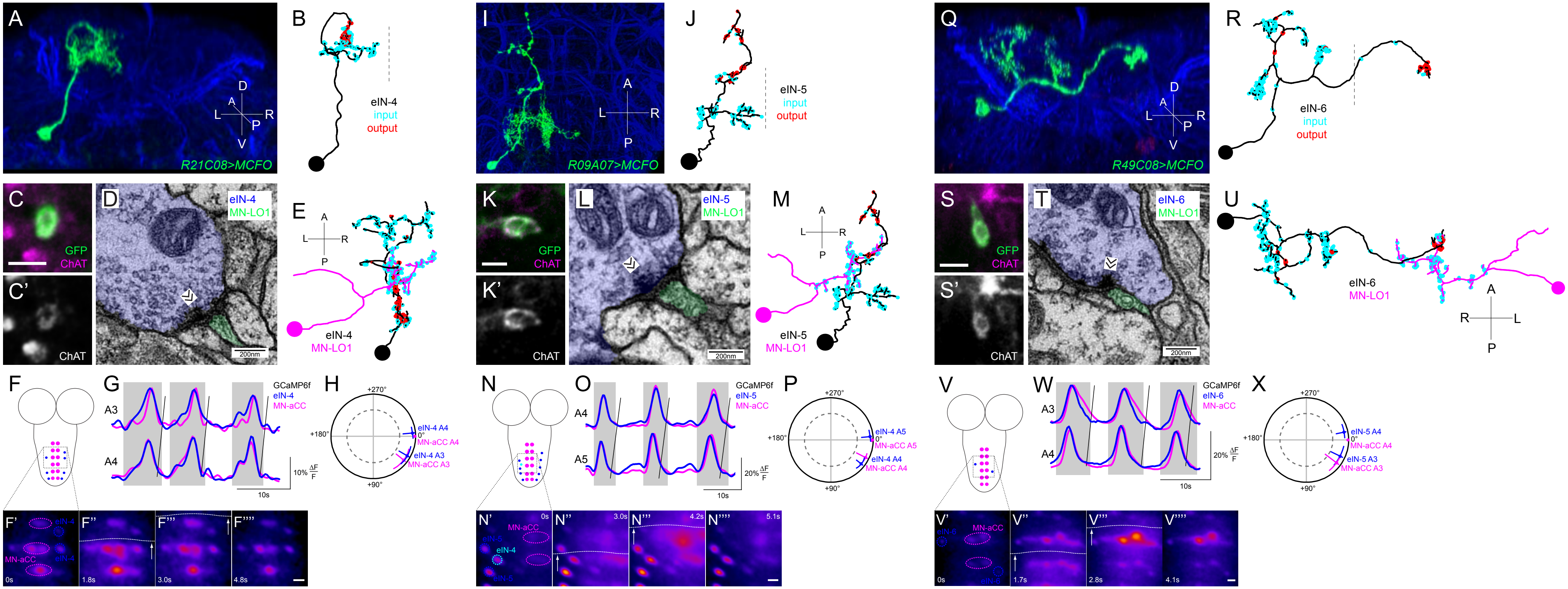

Figure S5, related to Figure 3. eIN-4, eIN-5 and eIN-6 innervate MN-LO1 and are recruited during locomotion.
Posterior (A, Q) and dorsal view (I) of light microscopy data and EM reconstructions (B, J, R) of eINs innervating MN-LO1. (C, K, S) Immunohistochemical labeling of SS01956>myrGFP (C), R09A07>myrGFP (K) and SS01404>myrGFP (S) showing pronounced ChAT staining. (D, L, T) Electron micrograph showing the apposition of eINs and MN-LO1, with synaptic vesicles and the presynaptic density clearly visible (chevrons). (E, M, U) Dorsal view of EM reconstructions of eINs innervating MN-LO1, either ipsilaterally (E, M) or contralaterally (U). (F-H, N-P, V-X) eINs show wave-like activity during fictive crawling. (F'-F'''', N'-N'''', V'-V''''). Stills showing GCaMP6f activity in eINs and aCC motoneurons as indicated in schematics (F, N, V), quantified in (G, O, W). (H, P, X) Coherency between eINs and aCC motor neurons in segment A3 and A4 or A4 and A5. Data are represented as mean ± 95% CI in (H), (P), and (X). n=5 for GCaMP imaging experiments, scale bars represent 10µm in (C, F''''), 5µm in (K, N'''', S, and V'''').

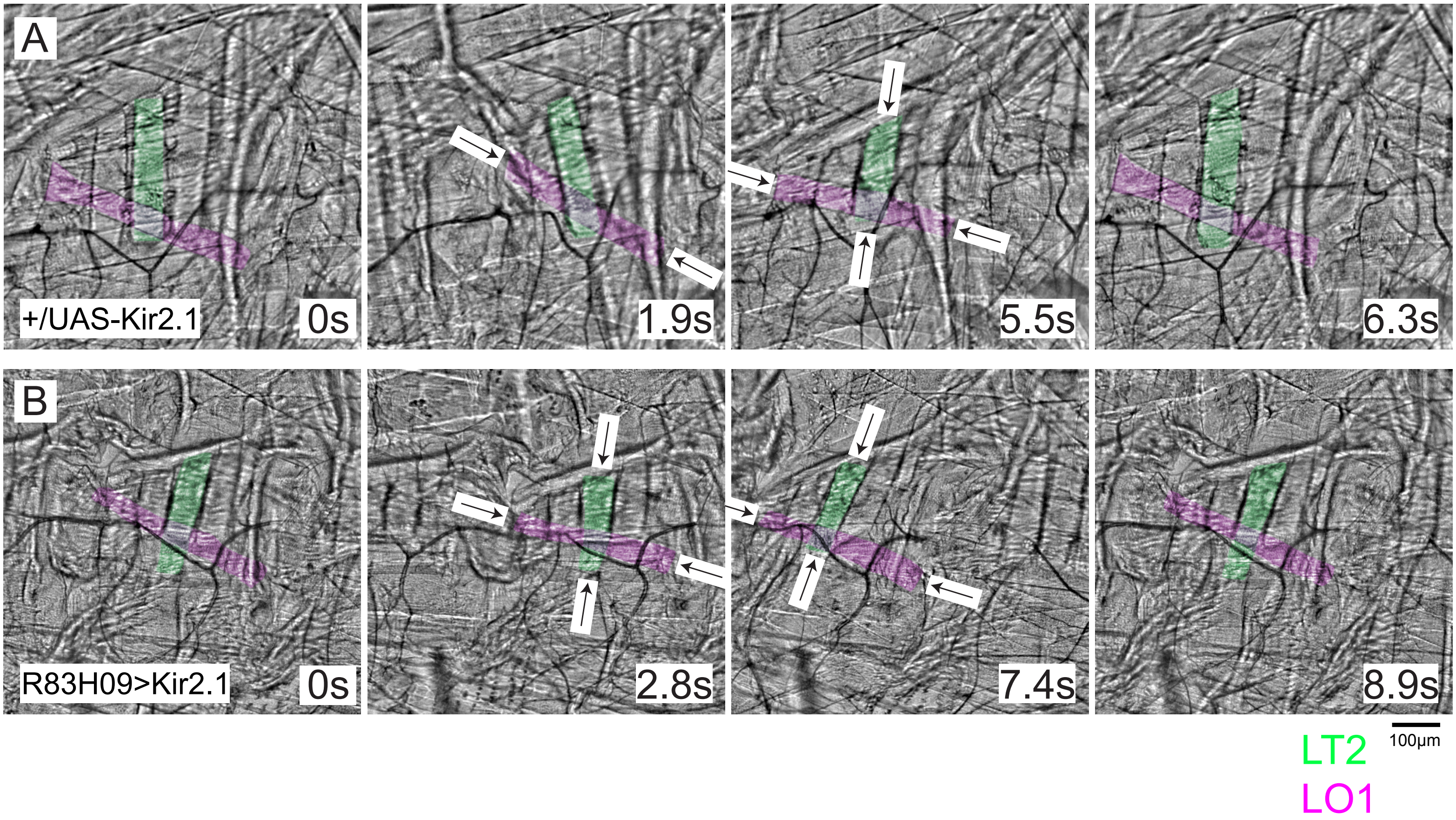

Figure S6, related to Figure 6. Inhibiting iIN-1 leads to a loss of the intrasegmental motor pattern. (A, B) Stills of imaging experiments to quantify muscle contraction patterns of LT2 (green) and LO1 (magenta) in a +/UAS-Kir2.1 control (A) and R83H09>Kir2.1 (B) preparation. Arrows indicate muscles contracting. See also Figure 6.
