# Supplementary material for: Selective Inhibition Mediates the Sequential Recruitment of Motor Pools
Source: Neuron. 2016 Aug 3;91(3):615–28. doi: 10.1016/j.neuron.2016.06.031 (PMC4980426; doi:10.1016/j.neuron.2016.06.031)
Supplement: Document S2. Article plus Supplemental Information [file mmc2.pdf]

Article

Neuron

Selective Inhibition Mediates the Sequential Recruitment of Motor Pools

Highlights

- *Drosophila* larval muscles are sequentially recruited during locomotion
- Motor neuron intrinsic properties do not determine order of recruitment
- EM reconstructions reveal divergent premotor networks of two sequential motor pools
- A $GABA^+$ neuron acts as a delay line mediating delayed motor pool activation

Authors

Maarten F. Zwart, Stefan R. Pulver, James W. Truman, Akira Fushiki, Richard D. Fetter, Albert Cardona, Matthias Landgraf

Correspondence

zwartm@janelia.hhmi.org (M.F.Z.), cardonaa@janelia.hhmi.org (A.C.)

In Brief

How locomotor systems generate overlapping sequences of local muscle contractions is unknown. Zwart et al. show in the *Drosophila* larva that an inhibitory neuron acts as a delay line to a specific population of motor neurons to generate such a sequence.

Zwart et al., 2016, Neuron *91*, 615–628
August 3, 2016 © 2016 The Authors. Published by Elsevier Inc.
http://dx.doi.org/10.1016/j.neuron.2016.06.031

CellPress

Selective Inhibition Mediates the Sequential Recruitment of Motor Pools

Maarten F. Zwart,[1,2,*] Stefan R. Pulver,[1,4] James W. Truman,[1] Akira Fushiki,[1] Richard D. Fetter,[1,5] Albert Cardona,[1,3,*] and Matthias Landgraf[2,3]
[1]HHMI Janelia Research Campus, Ashburn, VA 20147, USA
[2]Department of Zoology, University of Cambridge, Cambridge CB2 3EJ, UK
[3]Co-senior author
[4]Present address: School of Psychology and Neuroscience, University of St Andrews, St Andrews KY16 9JP, UK
[5]Present address: Department of Biochemistry and Biophysics, University of California, San Francisco, San Francisco, CA 94158, USA
*Correspondence: zwartm@janelia.hhmi.org (M.F.Z.), cardonaa@janelia.hhmi.org (A.C.)
http://dx.doi.org/10.1016/j.neuron.2016.06.031

SUMMARY

Locomotor systems generate diverse motor patterns to produce the movements underlying behavior, requiring that motor neurons be recruited at various phases of the locomotor cycle. Reciprocal inhibition produces alternating motor patterns; however, the mechanisms that generate other phasic relationships between intrasegmental motor pools are unknown. Here, we investigate one such motor pattern in the *Drosophila* larva, using a multidisciplinary approach including electrophysiology and ssTEM-based circuit reconstruction. We find that two motor pools that are sequentially recruited during locomotion have identical excitable properties. In contrast, they receive input from divergent premotor circuits. We find that this motor pattern is not orchestrated by differential excitatory input but by a GABAergic interneuron acting as a delay line to the later-recruited motor pool. Our findings show how a motor pattern is generated as a function of the modular organization of locomotor networks through segregation of inhibition, a potentially general mechanism for sequential motor patterns.

INTRODUCTION

Movements are generated by precise sequences of activity in motor systems. In spite of decades of research, the logic underlying the neural circuitry that produces these sequences during locomotion remains unclear (Büschges et al., 2011; Harris and Weinberg, 2012; McLean and Dougherty, 2015). Attempts to decipher this logic have largely focused on the alternating patterns of activity that underlie the recruitment of antagonistic motor units, such as flexors and extensors (Grillner, 2003; Grillner and Jessell, 2009; McLean and Dougherty, 2015; Talpalar et al., 2011; Tripodi et al., 2011), depressors and elevators (Burrows, 1996), and the bilaterally homologous motor units that generate left-right alternation (Grillner, 2003; Talpalar et al., 2013). A common circuit motif that underlies these antiphasic activity patterns are reciprocal inhibitory connections between premotor circuits (Büschges et al., 2011; Kiehn, 2011).

However, many movements require gradual, overlapping sequences of muscle contractions. For instance, synergistic motor pools are tuned across the entire phasic space during fictive locomotion in the mouse spinal cord (Hinckley et al., 2015; Machado et al., 2015) and fictive scratching in the turtle (Berkowitz and Stein, 1994), and many intrasegmental muscles in the cat contract sequentially with overlaps in their activation during various movements (Pratt et al., 1991). In spite of the prominence of this type of motor pattern, it is unknown how premotor circuits generate the required sequential patterns of activity within each segment in the appropriate motor neurons.

In principle, the sequential pattern can be established through two non-mutually exclusive mechanisms: first, a common source of interneuronal input could elicit temporally distinct responses in motor neurons that have different electrical properties (Johnson et al., 2005; Matsushima et al., 1993; Wang and McLean, 2014). Second, premotor networks could recruit motor units sequentially through differences in the delivery of excitatory or inhibitory input (Bagnall and McLean, 2014; Gabriel et al., 2011). In locomotor networks, motor neurons are ordered centrally to represent the spatial organization of their postsynaptic muscles, forming a myotopic map that also extends to their presynaptic partners (Landgraf et al., 2003; Okado et al., 1990; Romanes, 1964; Sürmeli et al., 2011; Tripodi et al., 2011). This conserved feature mediates the segregation of input onto different classes of motor neurons and could form the basis for the generation of different motor patterns.

In this study, we draw on the experimental advantages of the *Drosophila* larva to determine the neural basis for a motor pattern that is conceptually similar to the sequential pattern described in vertebrate motor systems. Specifically, we focus on delineating the circuit mechanisms underlying the generation of an intrasegmental sequence of overlapping contractions of two distinct muscle groups during larval crawling (Heckscher et al., 2012). First, using whole-cell electrophysiology, we show that motor neurons that innervate either muscle group do not differ in their intrinsic electrical properties, suggesting that their recruitment pattern must be the result of the organization of the presynaptic network. Second, reconstructions from serial section transmission electron microscopy (ssTEM) of

This is an open access article under the CC BY license (http://creativecommons.org/licenses/by/4.0/).

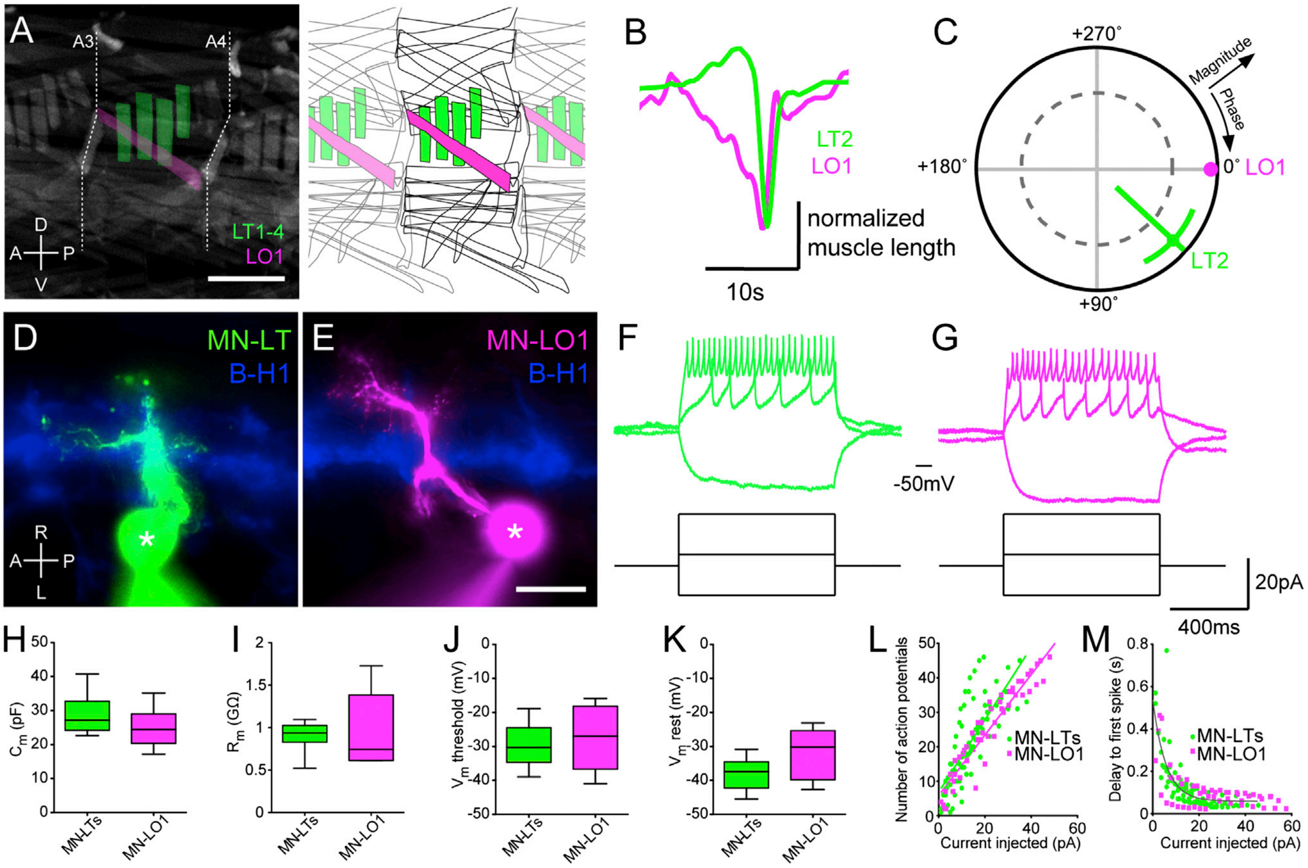

Figure 1. Motor Neuron Intrinsic Properties Do Not Contribute to the Generation of the Intrasegmental Motor Pattern Underlying Larval Crawling

(A) Longitudinal muscle LO1 (magenta) and transverse muscles LT1–LT4 (green) in a single segment of the *Drosophila* larva. Left panel shows GFP-labeled muscles of hemisegments A3–A5, schematized in the right panel. Scale bar, 200 μm.

(B) Contraction pattern of LT2 and LO1 in segment A4 in (A) during a crawling cycle.

(C) Polar plot of magnitude and phase of coherency of the two waveforms with LO1 as reference. Dashed line indicates $\alpha = 0.05$ for coherence magnitude statistically deviating from 0. Data are represented as mean ± 95% confidence interval (CI).

(D and E) Example motor neurons during patch-clamp recording from cell bodies (asterisks) labeled with Alexa Fluor 568 Hydrazide dye, pseudocolored green (D; MN-LT) or magenta (E; MN-LO1). Blue shading is mCD8::GFP expression under the *B-H1* promoter. Scale bar in (E), 5 μm.

(F and G) Example recordings of MN-LT (F) and MN-LO1 (G) during different levels of current injection.

(H–K) (H) Capacitance (C_m), (I) membrane resistance (R_m), (J) membrane voltage threshold to action potential (V_m threshold), and (K) resting membrane potential (V_m rest) of MN-LTs (green) and MN-LO1s (magenta). Boxplots show mean ± quartiles; whiskers minimum to maximum value. $p > 0.05$, t tests.

(L and M) The number of action potentials (L) and delay to first spike (M) as a function of the amplitude of current injection for MN-LTs (green) and MN-LO1s (magenta). There is no statistically significant difference between the slopes of the linear regression lines in (L) ($p > 0.05$), and one curve fits best the non-linear fit in (M). n = 9 for MN-LTs; n = 5 for MN-LO1. Also see Figure S1.

the premotor network show that motor neurons that are recruited at different phases of the intrasegmental locomotor cycle receive input from different sets of interneurons. This contrasts with functionally similar motor neurons, which share a high degree of common input. Third, probing further into the premotor network, we find that the motor pattern is not orchestrated by differential excitatory inputs but by a GABAergic inhibitory interneuron that specifically innervates the later-recruited class of motor neurons and acts as an intrasegmental delay line. Our results show that the segregation of input onto distinct intrasegmental motor neurons facilitates the generation of a widespread motor pattern through selective inhibition of a motor pool. This might represent a general mechanism for generating non-alternating phase relationships between intrasegmental motor pools.

RESULTS

Motor Neurons Innervating Functionally Distinct Muscles Have Similar Intrinsic Properties

Previous work established that locomotion in the *Drosophila* larva is mediated by peristaltic waves of muscle contractions, which, during forward locomotion, commence in posterior segments and propagate anteriorly from one segment to the next (Crisp et al., 2008). Within each segment, the longitudinal muscles, running parallel to the length of the animal, begin to contract

before transverse muscles, which are oriented perpendicular to the main body axis (Heckscher et al., 2012; Figures 1A and 1B). This is followed by a period of co-contraction of both muscle sets (Figures 1A and 1B). Therefore, this intrasegmental muscle contraction sequence is unlike alternating left-right or flexor-extensor activation, which has been a primary focus of studies in vertebrate model systems (Kiehn, 2011). This sequence is a signature of larval crawling in both first and third instar larvae (Heckscher et al., 2012; Pulver et al., 2015). The contractions represent highly coherent waveforms with contractions of transverse muscles occurring with an $\sim 42^\circ$ phase lag relative to longitudinal muscles during forward locomotion (Figure 1C). Importantly, the sequence is generated independently of sensory feedback (Pulver et al., 2015), ruling out an essential role of the musculature or proprioception. This motor pattern is therefore similar in concept to the sequential recruitment of synergistic intrasegmental motor pools in vertebrates.

We set out to study its neuronal basis. One underlying mechanism could be that the two sets of motor neurons that innervate longitudinal versus transverse muscles have different electrical properties, so that the same inputs would elicit temporally distinct responses (Choi et al., 2004; Gabriel et al., 2011; Schaefer et al., 2010; Wang and McLean, 2014). In order to test whether the motor neurons innervating the transverse muscles have intrinsic properties that delay their firing relative to motor neurons innervating longitudinal muscles, we performed whole-cell recordings in current-clamp and measured membrane voltages in response to steps and ramps of current injection in representative motor neurons (those innervating muscles lateral transverse 1–4 [MN-LT1–MN-LT4] and muscle lateral oblique 1 [MN-LO1], respectively; Figure 1). The membrane properties of these neurons were similar, with no statistical differences in membrane capacitance (C_m), input resistance (R_m), action potential threshold, or resting membrane potential (Figures 1H–1K; $p > 0.05$). Indeed, the number of action potentials fired in response to different steps of current injection was the same for the two representative groups (Figure 1L; $p > 0.05$). Crucially, there is no difference in the onset of firing in response to depolarizing current injection, as quantified by the delay to first spike (Figure 1M; $p > 0.05$). During rhythmic activity of the *Drosophila* larval motor network, the firing properties of motor neurons can be modulated by the action of the Na^+/K^+-ATPase in response to bursts of action potentials (Pulver and Griffith, 2010). However, we found that with rhythmic current injections the delay to first spike does not deviate between the two groups of motor neurons (Figures S1A and S1B, available online; $p > 0.05$). Furthermore, we could find no evidence of plateau potentials or rebound depolarizations in these cells (data not shown). Indeed, recording the action potentials these cells fire as the result of endogenous rhythmic excitatory input, we found no difference between the two groups of motor neurons in the duration between the onset of depolarization and the onset of firing (Figures S1C and S1D; $p > 0.05$). Taken together, these electrophysiological data suggest that the intrasegmental motor pattern is not mediated by differences in the intrinsic excitable properties of the output motor neurons. The data therefore point to divergence in premotor network input.

Functionally Distinct Motor Neurons Receive Divergent Input

Recent studies in vertebrate systems have suggested that functionally distinct motor units receive input from different complements of presynaptic neurons (Bagnall and McLean, 2014; Goetz et al., 2015; Stepien et al., 2010; Tripodi et al., 2011). Having established that the intrasegmental motor sequence in the *Drosophila* larva does not depend on the intrinsic properties of the output neurons, we next investigated the organization of the motor network presynaptic to representatives of the two different groups of motor neurons. To this end, we took advantage of an ssTEM volume of an entire first instar larval CNS, which is currently being reconstructed in a community-based effort (Fushiki et al., 2016; Heckscher et al., 2015; Ohyama et al., 2015). Within this ssTEM volume, we reconstructed in segment A1 MN-LT1–MN-LT4 as well as MN-LO1. These have the same axonal trajectory but distinct territories of dendritic arborization (Figure 2A). Next, we reconstructed the morphologies of all presynaptic partners of these motor neurons, a total of 198 arbors from thoracic, abdominal, and subesophageal segments (Figure S2; see Experimental Procedures for details). Out of 198 arbors, 111 different cell types could be identified based on morphology, providing 1,300 (92%) of the total of 1,409 input synapses onto the dendrites of both classes of motor neurons. Comparison of the complements of interneurons that are presynaptic to the two classes of motor neurons revealed a considerable degree of divergence between them (Figures 2B–2G). For example, MN-LT2 (representing a transverse-muscle motor neuron unit) and MN-LO1 (representing a longitudinal muscle-motor neuron unit) receive 82% of their input synapses from different presynaptic partners. In contrast, operationally similar motor neurons receive the vast majority of their input from common partners (e.g., 82% between MN-LT1 and MN-LT2). In order to determine the significance of this divergence in presynaptic partners, we compared the relative importance of the shared input between pairs of motor units: MN-LT1 and MN-LT2 versus MN-LT2 and MN-LO1. We find that presynaptic neurons that synapse onto two operationally similar motor neurons provide similar numbers of synapses to both (Figure 2F; Pearson's $r = 0.76$; $p < 0.0001$). In contrast, where the same presynaptic neuron forms synaptic connections with two operationally distinct motor neurons, there is no such correlation (Figure 2G; $p > 0.05$). In other words, functionally distinct motor neurons share few presynaptic partners; moreover, those that are shared either make few synaptic connections to both, or are more strongly connected to only one of them, further emphasizing the significance of the divergence of the presynaptic network. This circuit architecture suggests that the characteristic intrasegmental motor sequence could indeed be the result of the organization of the premotor network.

The Contribution of Premotor Excitatory Drive to the Motor Pattern

The distinct premotor circuits of the two classes of motor neurons could reflect a functional segregation of excitatory input, capable of delivering temporally distinct excitation. To test this hypothesis, we probed the premotor network to find cell types that could provide this excitation.

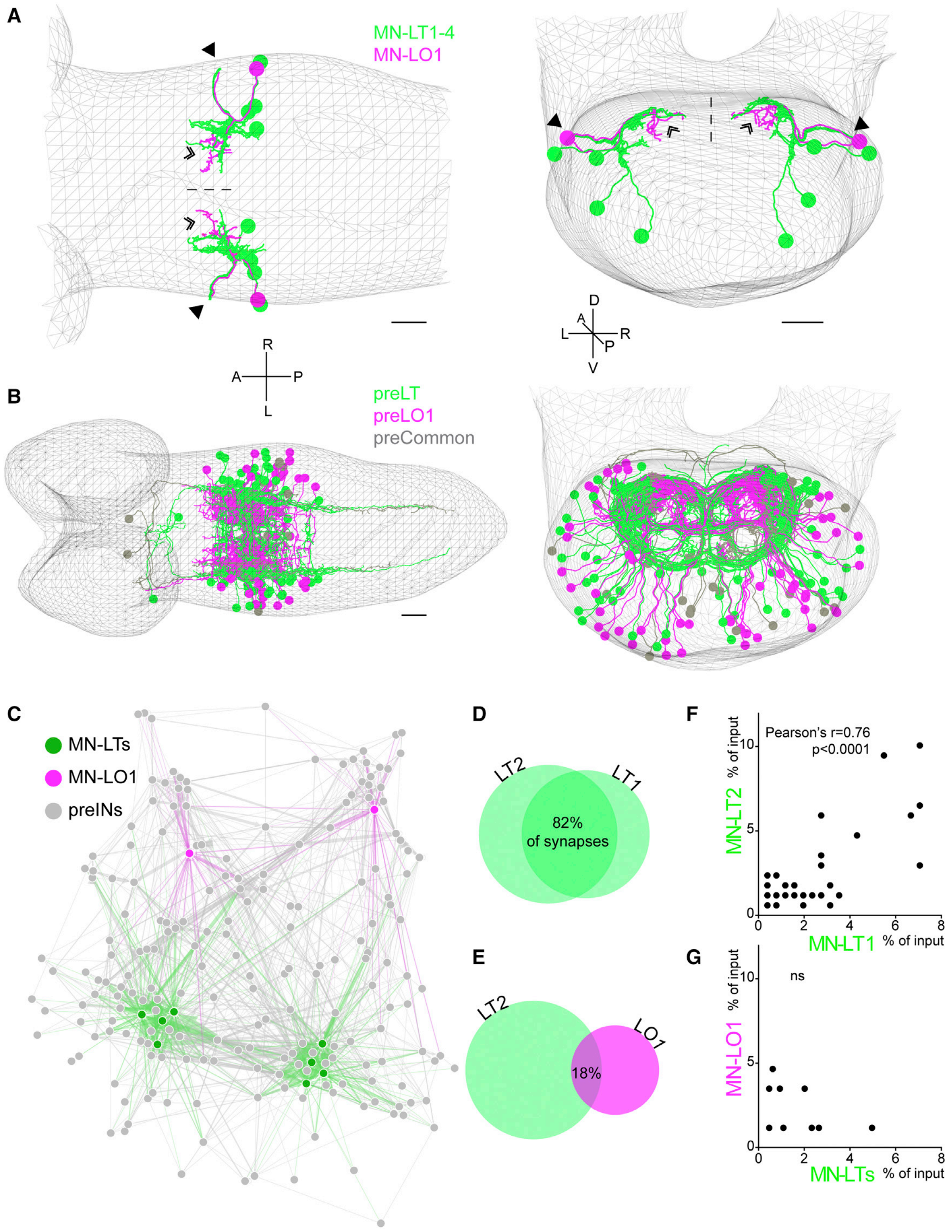

Figure 2. Functionally Distinct Motor Neurons Receive Divergent Input

(A) Dorsal (left) and posterior (right) views of the reconstructed motor neurons in segment A1 (MN-LTs in green; MN-LO1s in magenta), with efferents (arrowheads) and dendrites (chevrons) indicated. Mesh represents outline of the nervous system; dashed line indicates midline.

(B) Dorsal (left) and posterior (right) views of the reconstructed interneurons presynaptic to MN-LTs (green, "preLT"), MN-LO1s (magenta, "preLO1"), and both groups of motor neurons (gray, "preCommon"). Scale bars in (A) and (B), 10 μm.

(C) Force-directed network diagram showing reconstructed motor neurons and all of their presynaptic interneurons. The number of synapses between nodes determines the thickness of edges, which are color coded according to the identity of the postsynaptic node. In this graph, nodes similar in connectivity will be in close proximity. Motor neurons on the left side of the graph are from the left hemisegment of A1; those on the right are from the right hemisegment.

(legend continued on next page)

First, we identified *GAL4* driver lines that allow visualization of discrete sets of pre-motor interneurons (Li et al., 2014), as identified by ssTEM reconstructions. Next, we determined which of these interneuron types stained positive for the biosynthetic enzyme for the main excitatory neurotransmitter in this system, choline acetyltransferase (Baines et al., 1999). Among this subset we focused on those neurons that made more than 35 synaptic release sites onto the dendrites of the transverse-muscle motor neurons MN-LT1–MN-LT4 (>2.75% of total number of synaptic sites), but not onto MN-LO1. We thus identified three contralaterally projecting interneuron types (Figures 3 and S3), excitatory interneurons 1, 2, and 3 (eIN-1–eIN-3), derived from lineage 18/NB2-4 (eIN-1) and lineage 01/NB1-2 (eIN-2, eIN-3), respectively (Lacin and Truman, 2016). They are among the most strongly connected premotor interneurons within this premotor network, providing 14.6%, 13.5%, and 6.7% of total input synapses onto the transverse-muscle motor neurons MN-LT1–MN-LT4 per segment, respectively. Moreover, each of these three excitatory interneurons also synapses onto other motor neurons innervating other transverse muscles, such as MN-DT1.

To assess whether eIN-1–eIN-3 could play a role in setting the intrasegmental phase relationship between MN-LO1 and MN-LT1–MN-LT4 during larval crawling, we performed functional imaging of activity within these neurons. Specifically, we used a well-characterized fictive crawling activity paradigm, in which the nerve cord has been isolated from the periphery (Berni, 2015; Pulver et al., 2015; Experimental Procedures). Because there is no clean *GAL4* driver line for MN-LO1, we used the segmentally repeated aCC motor neuron as a robust indicator of fictive crawling phases and cycles (Figures 3G and S3; Pulver et al., 2015). MN-aCC is readily identifiable using *RRF-GAL4* (Fujioka et al., 2003), while MN-LO1 and the transverse-muscle motor neurons MN-LT1–MN-LT4 selectively express GAL4 in the *B-H1-GAL4* line (Garces et al., 2006; Sato et al., 1999). Using these reagents and paired whole-cell recording of their activity during fictive crawling, we established that, consistent with the fact that they both innervate longitudinal muscles, the MN-aCC and MN-LO1 motor neurons are active in phase during fictive locomotion (Figure S4).

We then measured fluorescence changes of the genetically encoded calcium indicator GCaMP6f (Chen et al., 2013) selectively expressed in a given eIN (see Experimental Procedures for details on driver lines) and the phase reference marker, MN-aCC. This experiment therefore allowed us to determine whether eIN-1, eIN-2, and eIN-3 are recruited during locomotion and to relate their activity to the activity pattern of the early recruited MN-aCC.

We found that all three eINs show wave-like activity during fictive locomotion (Figures 3G, 3H, and S3), with GCaMP6f dynamics highly coherent with those of MN-aCC. Unexpectedly, eIN activity is closest in phase with the early recruited MN-aCC located within the same segment (Figures 3I and S3). Therefore, these results do not support the hypothesis of sequential excitation generating the sequential intrasegmental motor pattern.

In order to further probe the role of excitation in the intrasegmental motor pattern, we decided to investigate the excitatory drive to the early recruited MN-LO1. This motor neuron receives input from many different cell types (a total of 70 arbors, providing a mean of 2.4 synapses each). We focused our efforts on the three most strongly connected cell types, which we named eIN-4–eIN-6. Collectively, eIN-4–eIN-6 provide 49 synapses (28.9% of MN-LO1 input) and, staining positive for choline acetyltransferase (Figure S4), are presumed excitatory. We characterized the activity patterns of these neurons during fictive locomotion. As before, we related the activity of eIN-4–eIN-6 to the activity of the segmentally repeated MN-aCC motor neuron by selectively expressing GCaMP6f both in a given eIN and in the phase reference marker MN-aCC (see Experimental Procedures for details on driver lines). We found that eIN-4–eIN-6 all show wave-like activity during fictive locomotion (Figure S4) and, similar to eIN-1–eIN-3, are highly coherent and closest in phase with the MN-aCC in the segment they innervate (Figure S5). These results indicate that the main excitatory premotor interneurons of both early recruited MN-LO1 and those of the later recruited MN-LTs have temporally similar activity patterns, in phase with MN-aCC. This strongly suggests that temporally distinct excitatory drive is unlikely to underlie the sequential motor pattern.

In order to further probe the role of the eINs in the generation of the motor pattern, we performed optogenetic stimulation of eIN-1–eIN-3, which are presynaptic to the MN-LTs. We selectively expressed *UAS-CsChrimson* (Klapoetke et al., 2014) in eIN-1–eIN-3, one cell type at a time, and assessed the effect of stimulating these neurons by measuring contractions of the transverse muscle LT2 and longitudinal muscle LO1 in a novel semi-intact preparation that exhibits the characteristic intrasegmental motor sequence (see Experimental Procedures). Acute, high-level stimulation (617 nm, 1.1 mW/mm^2) of eIN-1, eIN-2, or eIN-3 induces contraction of muscle LT2, but not muscle LO1 (Figures 3J and S3), suggesting that these neurons are indeed capable of driving the MN-LTs selectively and efficiently. Interestingly, chronic, low-level stimulation (617 nm, 0.01 mW/mm^2) of any of these eINs caused muscle LT2 to contract earlier than normal in the locomotor cycle, thus reducing the phase offset between LT2 and LO1 contractions (Figures 3K, 3L, and S3) ($p < 0.05$, Hotelling paired test, $n \geq 5$). This excitation level-dependent shift in the recruitment of MN-LTs suggests that during the normal locomotion cycle, a source of inhibition might selectively delay the recruitment of the MN-LTs.

The Intrasegmental Motor Sequence Depends on GABAergic or Glutamatergic Inhibition

In various other motor systems (Grillner and Jessell, 2009; Kiehn, 2011), inhibitory inputs generate alternating sequences

(D and E) Overlap in Venn diagrams is proportionate to the number of shared presynaptic partners, with percentage of total input synapses these partners provide indicated for functionally similar (D) and distinct (E) motor neurons.

(F and G) Pairwise comparison of relative synaptic contributions of shared presynaptic partners for functionally similar (F) and distinct (G) motor neurons. Also see Figure S2.

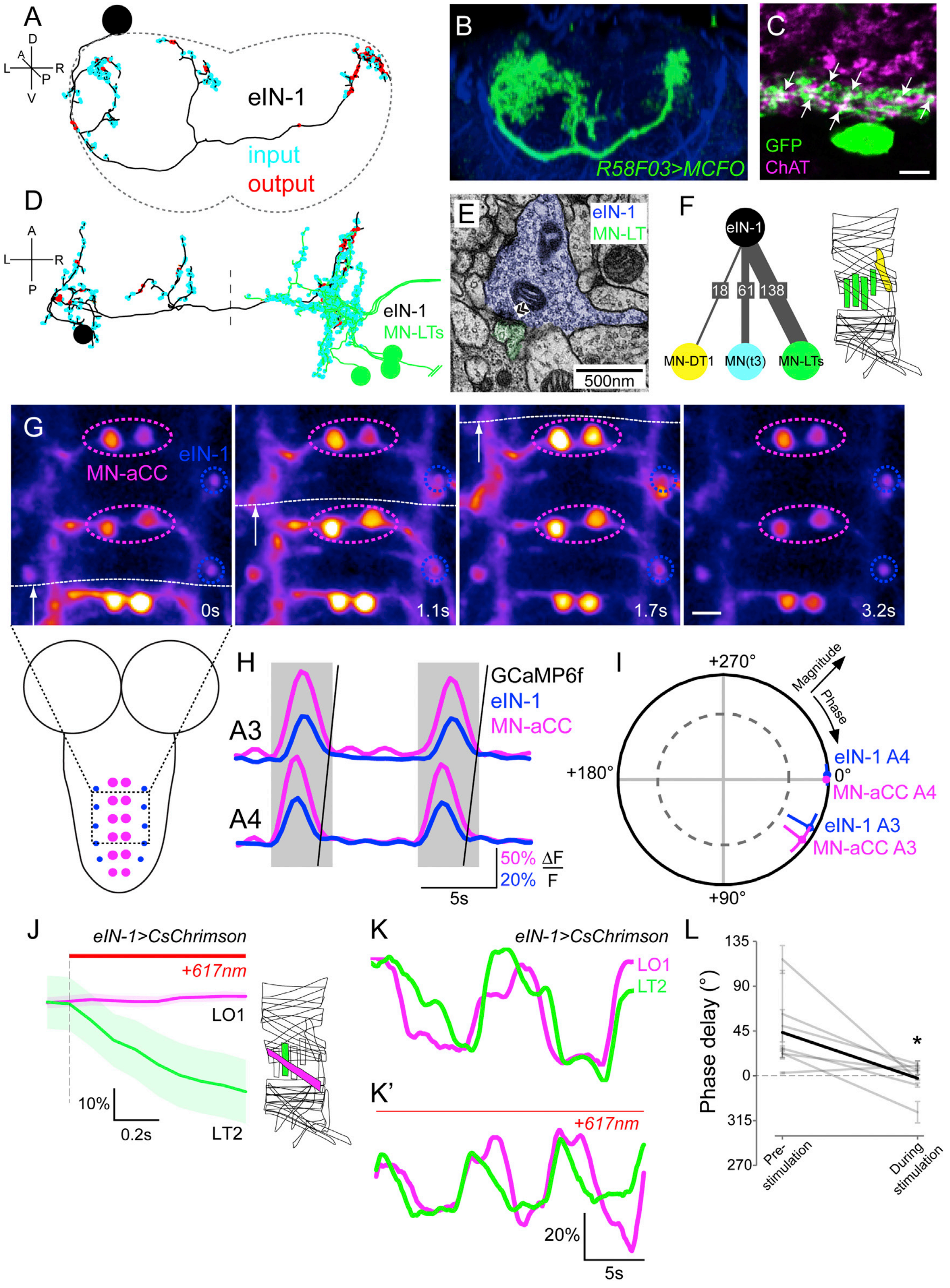

Figure 3. eIN-1 Innervates Transverse Motor Neurons and Is Recruited in Phase with Longitudinal Output in the Same Segment

(A and B) Posterior views of ssTEM reconstruction of eIN-1 (A) and light microscopy image of R58F03 > MCFO (see Experimental Procedures) (B).

(C) Single optical slice of *SS01970 > myrGFP* (expressing in eIN-1) showing pronounced ChAT staining in neurites (arrows).

(D) Dorsal view of an eIN-1 innervating the contralateral MN-LTs.

(E) Electron micrograph showing the apposition of eIN-1 and two MN-LTs, with presynaptic density indicated (chevrons).

(legend continued on next page)

of muscle activation. We reasoned that the observed segregation of input in our system may reflect differences in inhibition that underlie the pattern of activation of the two classes of motor neurons examined. We therefore performed muscle-imaging experiments in our semi-intact preparation (Figure 4A; see Experimental Procedures). We then bath-applied picrotoxin (PTX, 10^{-6} M; Figure 4B) to block glutamate and GABA-gated Cl^--channel-mediated inhibition (Liu and Wilson, 2013; Mauss et al., 2014; Rohrbough and Broadie, 2002). Whereas in control experiments the longitudinal and transverse muscle groups contract in sequence, we found that application of PTX effectively and selectively changes this motor pattern: while intersegmental waves remain intact, the longitudinal and transverse muscle groups within each segment now contract in synchrony (Figures 4C–4F; Hotelling paired test, $p < 0.01$, n = 5). This suggests that the motor network provides a source of PTX-sensitive inhibition that mediates the motor sequence.

A GABAergic Cell Type Presynaptic to One Class of Motor Neurons Is Required for the Motor Pattern

We reasoned that the source of the inhibition that generates the intrasegmental motor sequence likely resides within the network that is presynaptic to the later firing, transverse-muscle motor neurons. To test this hypothesis, we identified cells within the extensive premotor network that (1) contain GABA neurotransmitter, (2) exclusively innervate the transverse-muscle motor neurons, (3) are recruited during locomotion, and (4) are functionally required for the intrasegmental motor sequence.

First, we determined which of the premotor cell types found in our ssTEM reconstructions stained positive for the neurotransmitter GABA, and then selected those that made more than 35 synaptic release sites exclusively onto the dendrites of the transverse motor neurons (>2.75% of total number of synaptic sites; same threshold as for eINs). We thus identified three contralaterally projecting interneuron types (Figure 5; data not shown for inhibitory interneuron [iIN]-2 and iIN-3), iIN-1–iIN-3, which provide 2.8%, 15.1%, and 9.8% of total input synapses onto the transverse-muscle motor neurons per segment, respectively. Moreover, the majority of postsynaptic neurons of iIN-1–iIN-3 that could be identified are motor neurons with target muscles of similar orientation as muscles LT1–LT4 (Figure 5F). These three interneuron cell types therefore meet the first two selection criteria.

Next, to determine which of these iINs are recruited during locomotion, we performed functional imaging of neuronal activity as before. We found that only iIN-1, derived from abdominal lineage 14/NB4-1 (Lacin and Truman, 2016), shows wave-like activity during fictive locomotion (Figures 5G and 5H; data not shown for iIN-2 and iIN-3). iIN-1 GCaMP6f activity is highly coherent with that of MN-aCC, and is closest in phase to the aCC motor neuron located within the same segment (Figure 5I). Therefore, only iIN-1 fulfills all three criteria: it has a transmitter complement and activity profile consistent with it having the potential for introducing a delay in firing between longitudinal and transverse-muscle motor neurons.

To determine whether the activity of iIN-1 is required to generate the sequential intrasegmental motor pattern, we performed muscle-imaging experiments in animals in which we selectively inhibited the output of iIN-1 by expressing the hyperpolarizing potassium channel Kir2.1 (Baines et al., 2001). We found that targeting the expression of *UAS-Kir2.1* to iIN-1 using *R83H09-GAL4* interferes with the motor pattern: with each peristaltic wave, the intrasegmental sequence of muscle contractions that is normally observed is changed, so that now both muscle groups contract largely in synchrony (Figures 6A–6C; also see Figure S6; p = 0.003, n = 7). These results are consistent with our observation that the excitatory drive to the transverse-muscle motor neurons is in phase with activation of the longitudinal motor pool. We noticed that while *R83H09-GAL4* expresses in iIN-1 in all abdominal segments, it also expresses in other, as yet unidentified cell types in abdominal, thoracic, and subesophageal segments and the brain (Figure 6D). We therefore repeated the experiment using a more selective intersectional "split-GAL4" driver line, *SS01411-GAL4*, which expresses exclusively in iIN-1, though in a smaller number of abdominal segments (Figure 6D). The intrasegmental motor pattern defects seen with *SS01411-GAL4* targeted expression of *UAS-Kir2.1* were indistinguishable from those seen with *R83H09-GAL4* (Figure 6C; p = 0.004, n = 5). To corroborate the outcome of these experiments, we interfered with iIN-1 synaptic transmission in a different way, by targeting expression of *UAS-TeTxLC*, which prevents evoked neurotransmitter release (Sweeney et al., 1995). This has the same disruptive effect on the intrasegmental motor pattern as expressing Kir2.1 (Figure 6C; p = 0.0005, n = 6).

The data suggest that the activity of iIN-1 might act as a delay line to the transverse-muscle motor neurons and that this determines the intrasegmental motor pattern. If this is indeed the case, then, we reasoned, experimentally elevated levels of activity of iIN-1 should cause an enhanced phase shift between muscle contractions of LT2 versus LO1 during fictive crawling. To test this hypothesis, we optogenetically activated

(F) eIN-1 is presynaptic to MN-DT1 (yellow), which innervates a muscle of similar orientation as the MN-LTs; a motoneuron innervating an as yet unidentified muscle (cyan); as well as the MN-LTs (green). Included here are all connections of more than five synapses. Muscle diagram indicates identities of known target muscles.

(G and H) (G) Stills showing GCaMP6f activity in eIN-1 (blue dashed circles) and MN-aCC (magenta dashed circles) as indicated in schematic; quantified in (H). White arrow and dashed line in (G) indicate approximate front of peristaltic wave.

(I) Coherency between eIN-1 and MN-aCC in segments A4 and A3.

(J) Acute high-intensity optogenetic stimulation (617 nm, 1.1 mW/mm^2) of eIN-1 induces contraction specifically of transverse muscles.

(K and L) (K) Low-level chronic stimulation of eIN-1 (617 nm, 0.01 mW/mm^2) causes transverse muscles to contract earlier in the locomotor cycle; quantified in (L). Gray lines in (L) indicate individual preparations; black line represents mean. Hotelling paired test, $p < 0.05$ for (L).

n = 10 stimulations for (J), n = 5 animals for GCaMP imaging experiments, and n = 7 for (K) and (L). Data are represented as mean ± 95% CI in (I); mean ± SD in (J) and (L). Scale bar, 5 μm (C), 10 μm (G). See also Figures S3–S5.

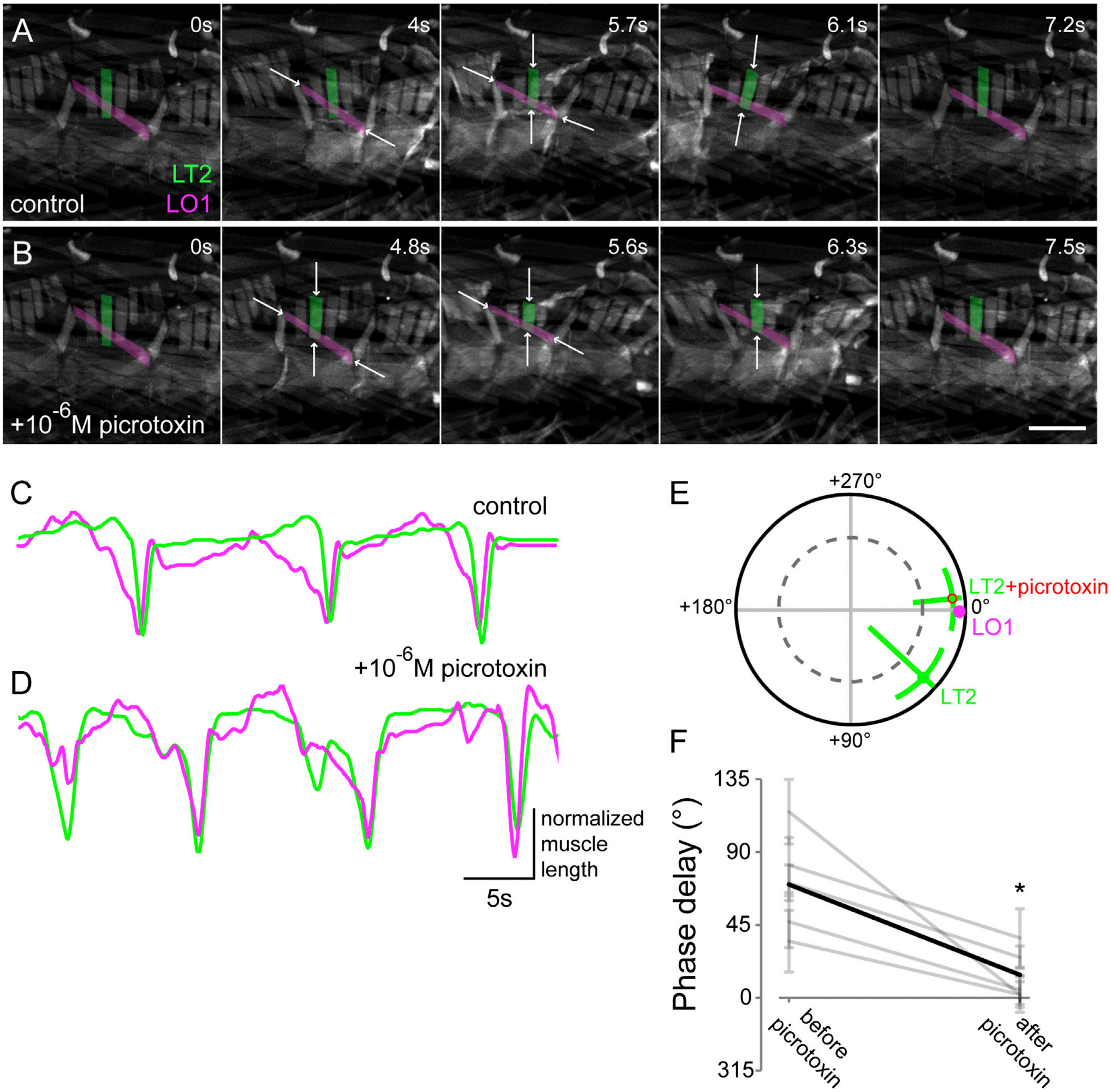

Figure 4. The Intrasegmental Motor Pattern Is Sensitive to PTX

(A–D) Muscle-imaging data showing contraction of muscles LT2 (green) and LO1 (magenta) during a single peristaltic wave before (A) and after (B) bath application of 10^{-6} M PTX; quantified in (C) and (D). Control data are the same as in Figure 1. Scale bar in (B), 200 μm. Arrows in (A) and (B) indicate muscles contracting.

(E) Coherency between muscles LT2 and LO1 before and after bath application of PTX in individual animals.

(F) Phase relationship between muscles LT2 and LO1 before and after bath application of PTX. Gray lines indicate individual preparations; black line represents mean. $p < 0.01$, Hotelling paired test. n = 5.

Data are represented as mean ± 95% CI in (E); mean ± SD in (F).

iIN-1 using *UAS-CsChrimson* expressed in iIN-1 with *R83H09-GAL4* and assessed the effect on the motor pattern during fictive crawling in our semi-intact preparation. Acute, high-level stimulation of iIN-1 (617 nm, 1.1 mW/mm^2) led to relaxation of muscle LT2 while leaving muscle LO1 unaffected (Figure 6E). Consistent with our hypothesis that iIN-1 acts as a delay line to the transverse-muscle motor neurons, low-level stimulation of iIN-1 (617 nm, 0.1 mW/mm^2) caused an increase in the

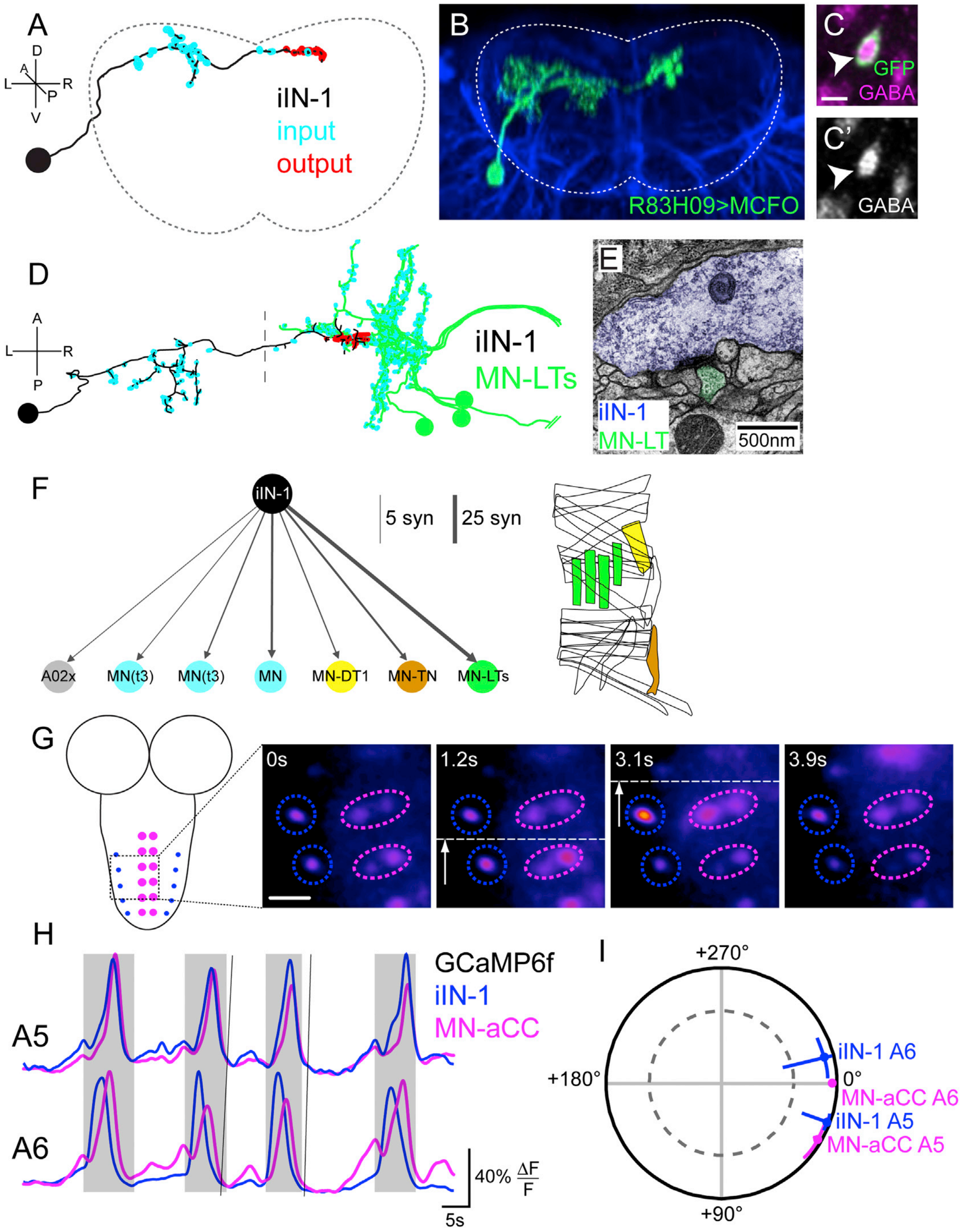

Figure 5. iIN-1 Specifically Innervates Transverse Motor Neurons and Shows Wave-like Activity during Fictive Locomotion

(A and B) Posterior view of ssTEM reconstruction (A) and light microscopy data (B) of iIN-1.

(C) Immunohistochemical labeling of *R83H09 > myrGFP* showing pronounced GABA staining.

(D) Dorsal view of an iIN-1 innervating the contralateral cluster of MN-LTs.

(E) Electron micrograph showing the apposition of iIN-1 and an MN-LT.

(F) iIN-1 is presynaptic to other motor neurons innervating muscles of similar orientation as the MN-LTs. Cyan motor neurons innervate unknown muscles; gray node indicates interneuron. Included in this diagram are all connections of more than five synapses. Muscle diagram indicates identity of known target muscles, color coded according to the left panel.

(G and H) (G) Stills showing GCaMP6f activity of iIN-1 and aCC motor neurons as indicated in schematic; quantified in (H).

(I) Coherency between iIN-1 and aCC motor neurons in segments A5 and A6. Data are represented as mean ± 95% CI, n = 5.

Scale bar, 5 μm (C), 10 μm (G).

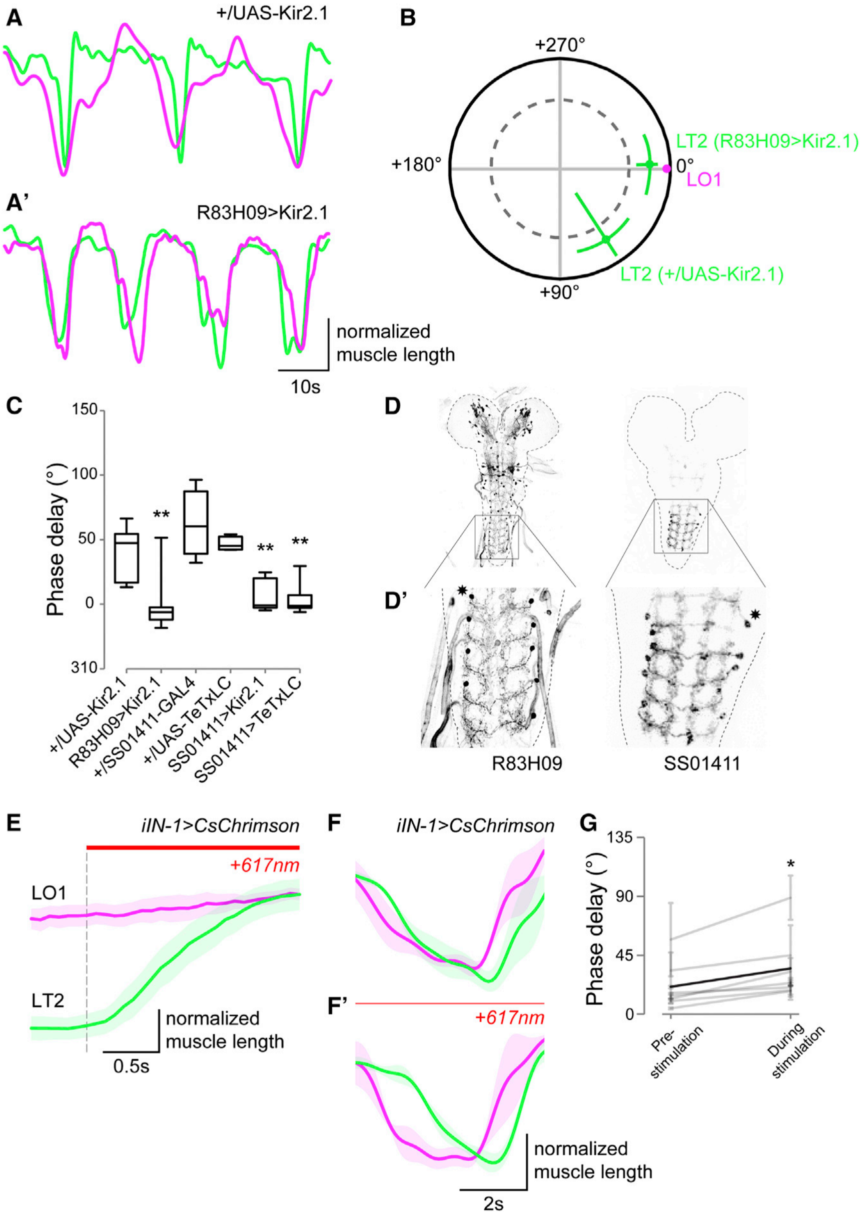

Figure 6. The Output of iIN-1 Is Required to Generate the Intrasegmental Motor Pattern

(A and B) Contraction of muscles LT2 (green) and LO1 (magenta) in a *+/UAS-Kir2.1* control animal (A) and an *R83H09* > *Kir2.1* animal (A′), the coherency between which is quantified in (B).

(C) Phase relation between muscles LT2 and LO1 for various genotypes tested. Pairwise Watson-Williams test, p = 0.003, p = 0.004, and p = 0.0005 for *R83H09* > *Kir2.1*, *SS01411* > *Kir2.1*, and *SS01411* > *TeTxLC*, respectively (n ≥ 5). Boxplots show mean ± quartiles; whiskers minimum to maximum value.

(D) Expression patterns of GAL4 drivers used in this experiment, enlarged in (D′). Asterisks indicate example cell bodies.

(E) Acute high-intensity optogenetic stimulation (617 nm, 1.1 mW/mm^2) of iIN-1 induces specific relaxation of the transverse muscles. Mean ± SEM of ten trials.

(F) Low-level chronic stimulation of iIN-1 (617 nm, 0.1 mW/mm^2) causes transverse muscles to contract later in the locomotor cycle. Mean ± SEM of ten consecutive contractions of muscles LO1 and LT2 in the same animal (F) pre-stimulation and (F′) during stimulation.

(G) The phase delay between muscle LO1 and LT2 contractions is enhanced in response to low-level chronic stimulation of eIN-1 (617 nm, 0.1 mW/mm^2). Gray lines indicate individual preparations ± SD; black line represents mean. p < 0.05, Hotelling paired test, n = 7.

See also Figure S6.

phase shift between muscles LO1 and LT2 (Hotelling paired test, p < 0.05, n = 7). Taken together, our results suggest that the intrasegmental phase relationship between the longitudinal and transverse motor units is set by the subset-specific iIN-1. Moreover, iIN-1 seems to act as a delay line that modulates the effects of coincidental excitation to both motor pools.

DISCUSSION

The circuit mechanisms that generate movements have been studied for many decades, in large part focusing on the alternating contractions of antagonistic muscles such as flexors and extensors (Büschges et al., 2011; Goulding, 2009; Kiehn, 2011; Miri et al., 2013). However, many motor pools are recruited sequentially, in largely overlapping patterns of activity (Berkowitz and Stein, 1994; Hinckley et al., 2015; Machado et al., 2015; Pratt et al., 1991). In this study, we investigate the neural mechanisms of such a pattern, focusing on an intrasegmental sequence of muscle contractions that is characteristic for larval crawling. Working with the *Drosophila* larva, we demonstrate that motor neurons that are recruited at different phases of the intrasegmental locomotor cycle receive largely divergent input and that the activity of an identified inhibitory interneuron is required for generating the phase delay.

Intrinsic Excitable Properties and the Recruitment of Motor Neurons

The output of a neural network is shaped by the intrinsic properties of its constituent neurons. For instance, the biophysical properties of different motor neuron populations in part determine their differential recruitment in the zebrafish spinal cord (Gabriel et al., 2011; McLean et al., 2007). In the *Drosophila* larva, a delay to action potential firing is mediated by a *Shal*-encoded I_A current in the RP2 motor neuron (Choi et al., 2004; Schaefer et al., 2010). Focusing on the motor neurons that are sequentially recruited during larval crawling, we found no evidence of

differences in their electrical properties. Instead, we found that the sequential intrasegmental recruitment is due to differences in the synaptic input that these different motor units receive.

Segregation of Premotor Connectivity

For many sensory systems, axon terminals are arranged in the CNS to form neural representations of sensory neuron modality and topography (Fitzpatrick and Ulanovsky, 2014). This straightforward link between neuronal anatomy and function has been less clear in motor systems. In the mouse spinal cord, the dorsal-ventral segregation of motor pools pre-figures sensory-motor connectivity (Sürmeli et al., 2011), and largely spatially segregated sets of interneurons connect to antagonistic motor neurons that innervate flexor and extensor muscles in the mouse (Tripodi et al., 2011).

Here, we characterized with single-synapse resolution the premotor circuitry of operationally different motor neurons in the *Drosophila* larva by electron microscopy (EM)-based reconstructions. This allowed us to establish that the myotopic organization of motor neurons is accompanied by a similarly segregated divergence of their presynaptic inputs: functionally similar motor neurons share many of their presynaptic partners (34/75 [45%] for MN-LT1 and MN-LT2), whereas functionally distinct motor neurons share few (9/112 [8%] between MN-LT2 and MN-LO1). Moreover, functionally similar motor neurons receive the majority of their synaptic input from shared presynaptic partners (82% of synapses provided by 45% of all presynaptic cells). In contrast, the few presynaptic partners that are shared between operationally distinct motor neurons are generally connected more strongly to one, or weakly to both, type of motor neuron.

As a note of caution, in our EM analysis, given previous evidence, we assumed that synapse number positively correlates with synapse strength. First, the number of synapses between two cells in this system was found to positively correlate with the responsiveness of the postsynaptic cell to presynaptic stimulation (Ohyama et al., 2015). Second, at the larval neuromuscular junction the strength of the postsynaptic response also correlates with synapse number (Budnik and Ruiz-Canada, 2006; Büschges et al., 2011; McLean and Dougherty, 2015). Third, we found little variability in the size of pre- and postsynaptic densities within the CNS of the *Drosophila* larva (M.F.Z. and A.C., unpublished data), in marked contrast to synapses in mammals, which can range in size over several orders of magnitude (Harris and Weinberg, 2012; Talpalar et al., 2011; Tripodi et al., 2011). These strands of evidence suggest that the number of synapses between central neurons likely correlates with the physiological relevance of connections.

Divergent Input and the Generation of Different Motor Patterns

It has been proposed that alternating muscle contractions are generated by largely divergent sets of premotor neurons, providing the antiphasic rhythmic drive through reciprocal inhibitory interactions (Grillner, 2003; Kiehn, 2011; Talpalar et al., 2011). It has been unclear how more gradual, overlapping sequences of muscle contractions, which are common to most movements, are generated (Bellardita and Kiehn, 2015; Berkowitz and Stein, 1994; Hinckley et al., 2015; Machado et al., 2015; Pratt et al., 1991). In the zebrafish, different groups of motor neurons are incrementally recruited with increasing swimming speeds by distinct sub-populations of V2a excitatory interneurons (Ampatzis et al., 2014; Gabriel et al., 2011; McLean et al., 2008). In the larval *Drosophila* motor network, we found that sequentially recruited groups of motor neurons receive input from different complements of interneurons. Unexpectedly, we found that the sets of excitatory premotor interneurons that innervate the early and late-acting motor pools are recruited in phase. Instead, we found that the sequential motor pool recruitment is generated by the GABAergic premotor interneuron iIN-1, which selectively innervates the later recruited MN-LTs. Furthermore, chronic, low-level optogenetic stimulation of this inhibitory neuron caused the MN-LTs to be recruited later in the locomotor cycle, while low-level stimulation of the eINs presynaptic to MN-LTs caused their earlier recruitment. Our data are compatible with a model in which the balance between excitation and inhibition shapes the phase delay, with the iIN-1 in effect acting as a delay line for the later recruited transverse-muscle motor neurons. An obvious functional implication of the segregated and diversified architecture is an inherent capacity for generating distinct motor patterns by differentially recruiting premotor elements, thereby mediating the ability to perform the diverse movements underlying the animal's behavioral repertoire. For example, one could envisage how selective recruitment of iIN-1 could mediate a switch from a behavior in which the longitudinal and transverse muscles contract in sequence (e.g., crawling) to another in which they co-contract. In this light, it will be interesting to see whether similar segregated sources of inhibition mediate the generation of gradual sequences of muscle contractions in other systems, such as those innervating synergistic muscles in vertebrates (Bikoff et al., 2016; Goetz et al., 2015; Laine et al., 2015; Tripodi et al., 2011).

Conclusions

We have identified a circuit motif embedded in the myotopic map that generates the sequential contraction of two muscle groups, which is characteristic for crawling in *Drosophila* larvae. Our findings on the segregated premotor circuitry are consistent with reports from mouse and zebrafish (Bagnall and McLean, 2014; Tripodi et al., 2011), suggesting that their last common ancestor contained a modular motor system that evolved to support the axial and limb networks that allow for the differential control of muscles (Büschges et al., 2011). Similar circuit motifs may be responsible for sequential motor patterns manifest in many behaviors across the animal kingdom.

EXPERIMENTAL PROCEDURES

Animal Rearing and Fly Strains

All animals were raised at 25°C on standard cornmeal-based food, supplemented with all-trans retinal (1 mM) in the case of optogenetic stimulation experiments. First instar larvae were used in the ssTEM data; feeding third instar larvae were used for all other experiments. We used the following genotypes: *w⁻;+;B-H1-GAL4* (Sato et al., 1999) crossed to *UAS-mCD8::GFP* animals for electrophysiology; *w⁻;UAS-GCaMP6f; RRF-GAL4* (Chen et al., 2013; Fujioka et al., 2003) crossed to *w⁻;R83H09-GAL4* or *w⁻;R09A07-GAL4* from the Rubin collection, or the split-GAL4 drivers (Luan et al., 2006; Pfeiffer

et al., 2010) *SS01956-GAL4*, *SS01404-GAL4*, *SS01379-GAL4*, *SS02056-GAL4*, *SS01411-GAL4*, and *SS01970-GAL4*, based on the Rubin collection for GCaMP6f imaging; the muscle marker line *w⁻;G203;ZCL2144* (Crisp et al., 2008) for Figure 4; *w⁻;UAS-Kir2.1* (Baines et al., 2001) and *w⁻;UAS-TeTxLC* (Sweeney et al., 1995) to inhibit neural activity; *w⁻;UAS-CsChrimson::mVenus* (Klapoetke et al., 2014) crossed to the appropriate *GAL4* driver lines for optogenetic stimulation. The "FLP-out" approach for stochastic single-cell labeling (MCFO) has been described in detail elsewhere (Nern et al., 2015).

Reconstruction of Premotor Circuits Using ssTEM Data

ssTEM data were analyzed as described in Ohyama et al. (2015). Motor neurons were identified and reconstructed within the ssTEM volume based on their axonal projection patterns (all MN-LTs and MN-LO1 assessed here project through segmental nerve a [SNa]; Landgraf et al., 1997), cell body position, and dendritic morphologies (M.L. and J. Lupton, unpublished data). All synapses onto these motor neurons were annotated and used to identify and reconstruct all presynaptic partners.

Electrophysiology

All electrophysiology experiments were performed as described in Marley and Baines (2011). The fluorescent dye Alexa Fluor 568 Hydrazide (100 μM, ThermoFisher Scientific) was added to the intracellular solution to aid identification of patched neurons. Data were collected with a multi-clamp 700B amplifier and digitized at 10 kHz using a Digidata 1550 (both Molecular Devices). Recordings were analyzed using custom scripts in Spike2 (Cambridge Electronic Design).

Immunohistochemistry

Immunohistochemistry was performed as described in Li et al. (2014). We dissected out larval CNSs as described before (Zwart et al., 2013), and fixed them in 4% paraformaldehyde for 30 min at room temperature to stain for GABAergic interneurons, or in Bouin's fixative for 5 min at room temperature to stain for cholinergic interneurons. Antibodies used were polyclonal anti-GABA antibody (Sigma-Aldrich; 1:200) or monoclonal ChAT-4B1 antibody (DSHB Hybridoma Product ChAT4B1, deposited to the DSHB by Salvaterra, P.M.; 1:100). Images were taken with a 710 laser-scanning confocal microscope (Zeiss) using a 20×/0.8 NA objective and contrast adjusted using Fiji software (Schindelin et al., 2012).

Calcium Imaging

For all calcium imaging experiments, we used a 488 nm diode laser (Thorlabs) in conjunction with a spinning disk confocal imager (Crest X-Light) mounted on an Olympus BX51WI microscope. We collected images at 5–10 Hz with an Andor iXon Ultra 897 EMCCD camera (Andor Technologies) using Winfluor software (John Dempster, University of Strathclyde), which was also used to drive the piezo controller (Physik Instrumente) moving the objective (Olympus, 20X/1.0 NA) for generating z stacks. Custom MATLAB scripts were used to measure and extract changes in fluorescence in regions of interest. Optical signals were then visualized and analyzed in Fiji, MATLAB, and Spike2.

Live Imaging of Muscle Activity

We developed a semi-intact preparation to record contractions of muscles with reduced sensory feedback. Third instar larvae were dissected as in Pulver and Griffith (2010), but two to three segmental nerve roots were left intact. We loosely pinned the preparation to a Sylgard-covered dish. Individual muscle contractions within innervated segments were then imaged using a 10× objective on an Olympus BX51WI microscope. The aperture of the field diaphragm was reduced to ensure the nervous system was not illuminated. The posterior and anterior attachment points of LO1 (also known as m5), as well as the medial and lateral attachment points of LT2 (also known as m22) were tracked using the manual tracking plugin (Fiji). Muscle length was calculated and used as a measure of muscle activation. In a subset of experiments, we applied 10^{-6} M PTX (Sigma-Aldrich) to preparations by manually exchanging the bath solution with a Pasteur pipette. For optogenetic stimulation experiments, 617 nm light provided by an OptoLED light source (Cairn) was delivered onto the preparation through the objective.

Coherence Analysis of Periodic Activity

To determine the phase relationship between periodic signals in calcium-imaging and muscle-imaging experiments, we used direct multi-taper estimates of power spectra and coherency (Cacciatore et al., 1999; Percival and Walden, 1993; Pulver et al., 2015; Taylor et al., 2003). In all experiments, we first performed a fast Fourier transform of the reference waveform (either the LO1 muscle or MN-aCC) in order to determine its spectral composition. We then determined the frequency at which the reference signal had the greatest power (the "dominant" frequency) and compared the coherence and phase relationship at that particular frequency between the reference signal and the other muscles or neurons, as appropriate. This analysis can efficiently compare the phase relationships between relatively complex waveforms, while attaching less weight to the peaks of activity, which are generally less informative in this context. Estimates were calculated with a time-bandwidth product of five and seven tapers. All spectral calculations were carried out using custom scripts written in MATLAB, now freely available online (https://github.com/JaneliaSciComp/Groundswell).

Statistics

Throughout the text, values are given in mean ± SE unless otherwise stated. We tested data for normality using the Shapiro-Wilk test, with a = 0.05. When data were normally distributed, t tests were used to test for significant differences. Otherwise, two-sample Wilcoxon tests were used. Linear regression, non-linear fitting of curves, and correlation analyses were performed in Prism (GraphPad Software); angular statistical analyses of results obtained with coherency analysis were carried out in Oriana. $p < 0.05$ was considered statistically significant in all experiments.

SUPPLEMENTAL INFORMATION

Supplemental Information includes six figures and can be found with this article online at http://dx.doi.org/10.1016/j.neuron.2016.06.031.

AUTHOR CONTRIBUTIONS

M.F.Z. devised the project, co-wrote the manuscript, and performed all experiments and analyses and most EM reconstructions. S.R.P. co-wrote the manuscript, developed the semi-intact preparation, and contributed to the muscle-imaging experiments. J.W.T. characterized expression of GAL4 driver lines and is responsible for the identification of most larval cell types. A.F. contributed to reconstructions. R.D.F. performed the sample preparation, serial sectioning and electron microscopy imaging for the ssTEM dataset. A.C. and M.L. co-wrote the manuscript and supervised the project.

ACKNOWLEDGMENTS

We would like to thank Casey Schneider-Mizell for help training M.F.Z. in EM reconstruction, generating Figure 2C, and contributing to EM reconstructions; Karen Hibbard and other members of the fly facility; Adam Taylor from Janelia Scientific Computing and William Rowell from Janelia Technical Resources for technical assistance; and Aref Arzan Zarin, Ingrid Andrade, and Philipp Schlegel for their contributions to EM reconstructions. We thank the Fly EM Project Team for assisting R.D.F. with imaging the serial sections of the larval central nervous system with transmission electron microscopy. We would also like to thank Gerry Rubin and Marta Zlatic for contributing GAL4 lines and Misha Ahrens, Vivek Jayaraman, Gareth Miles, and Ellie Heckscher for valuable comments on the manuscript. This work was supported by the Howard Hughes Medical Institute, the HHMI Janelia Visitor Program (M.F.Z. and M.L.), an Isaac Newton Trust/ISSF Wellcome Trust, and a Wellcome Trust grant (092986/Z) to M.L.

Received: September 24, 2015
Revised: March 22, 2016
Accepted: June 8, 2016
Published: July 14, 2016; corrected online August 3, 2016

REFERENCES

Ampatzis, K., Song, J., Ausborn, J., and El Manira, A. (2014). Separate microcircuit modules of distinct v2a interneurons and motoneurons control the speed of locomotion. Neuron *83*, 934–943.

Bagnall, M.W., and McLean, D.L. (2014). Modular organization of axial microcircuits in zebrafish. Science *343*, 197–200.

Baines, R.A., Robinson, S.G., Fujioka, M., Jaynes, J.B., and Bate, M. (1999). Postsynaptic expression of tetanus toxin light chain blocks synaptogenesis in Drosophila. Curr. Biol. *9*, 1267–1270.

Baines, R.A., Uhler, J.P., Thompson, A., Sweeney, S.T., and Bate, M. (2001). Altered electrical properties in Drosophila neurons developing without synaptic transmission. J. Neurosci. *21*, 1523–1531.

Bellardita, C., and Kiehn, O. (2015). Phenotypic characterization of speed-associated gait changes in mice reveals modular organization of locomotor networks. Curr. Biol. *25*, 1426–1436.

Berkowitz, A., and Stein, P.S. (1994). Activity of descending propriospinal axons in the turtle hindlimb enlargement during two forms of fictive scratching: broad tuning to regions of the body surface. J. Neurosci. *14*, 5089–5104.

Berni, J. (2015). Genetic dissection of a regionally differentiated network for exploratory behavior in Drosophila larvae. Curr. Biol. *25*, 1319–1326.

Bikoff, J.B., Gabitto, M.I., Rivard, A.F., Drobac, E., Machado, T.A., Miri, A., Brenner-Morton, S., Famojure, E., Diaz, C., Alvarez, F.J., et al. (2016). Spinal inhibitory interneuron diversity delineates variant motor microcircuits. Cell *165*, 207–219.

Budnik, V., and Ruiz-Canada, C. (2006). The Fly Neuromuscular Junction: Structure and Function (Elsevier).

Burrows, M. (1996). The Neurobiology of an Insect Brain (Oxford University Press).

Büschges, A., Scholz, H., and El Manira, A. (2011). New moves in motor control. Curr. Biol. *21*, R513–R524.

Cacciatore, T.W., Brodfuehrer, P.D., Gonzalez, J.E., Jiang, T., Adams, S.R., Tsien, R.Y., Kristan, W.B., Jr., and Kleinfeld, D. (1999). Identification of neural circuits by imaging coherent electrical activity with FRET-based dyes. Neuron *23*, 449–459.

Chen, T.-W., Wardill, T.J., Sun, Y., Pulver, S.R., Renninger, S.L., Baohan, A., Schreiter, E.R., Kerr, R.A., Orger, M.B., Jayaraman, V., et al. (2013). Ultrasensitive fluorescent proteins for imaging neuronal activity. Nature *499*, 295–300.

Choi, J.C., Park, D., and Griffith, L.C. (2004). Electrophysiological and morphological characterization of identified motor neurons in the Drosophila third instar larva central nervous system. J. Neurophysiol. *91*, 2353–2365.

Crisp, S., Evers, J.F., Fiala, A., and Bate, M. (2008). The development of motor coordination in Drosophila embryos. Development *135*, 3707–3717.

Fitzpatrick, D., and Ulanovsky, N. (2014). Editorial overview: neural maps. Curr. Opin. Neurobiol. *24*, iv–vi.

Fujioka, M., Lear, B.C., Landgraf, M., Yusibova, G.L., Zhou, J., Riley, K.M., Patel, N.H., and Jaynes, J.B. (2003). Even-skipped, acting as a repressor, regulates axonal projections in Drosophila. Development *130*, 5385–5400.

Fushiki, A., Zwart, M.F., Kohsaka, H., Fetter, R.D., Cardona, A., and Nose, A. (2016). A circuit mechanism for the propagation of waves of muscle contraction in Drosophila. eLife *5*, e13253.

Gabriel, J.P., Ausborn, J., Ampatzis, K., Mahmood, R., Eklöf-Ljunggren, E., and El Manira, A. (2011). Principles governing recruitment of motoneurons during swimming in zebrafish. Nat. Neurosci. *14*, 93–99.

Garces, A., Bogdanik, L., Thor, S., and Carroll, P. (2006). Expression of Drosophila BarH1-H2 homeoproteins in developing dopaminergic cells and segmental nerve a (SNa) motoneurons. Eur. J. Neurosci. *24*, 37–44.

Goetz, C., Pivetta, C., and Arber, S. (2015). Distinct limb and trunk premotor circuits establish laterality in the spinal cord. Neuron *85*, 131–144.

Goulding, M. (2009). Circuits controlling vertebrate locomotion: moving in a new direction. Nat. Rev. Neurosci. *10*, 507–518.

Grillner, S. (2003). The motor infrastructure: from ion channels to neuronal networks. Nat. Rev. Neurosci. *4*, 573–586.

Grillner, S., and Jessell, T.M. (2009). Measured motion: searching for simplicity in spinal locomotor networks. Curr. Opin. Neurobiol. *19*, 572–586.

Harris, K.M., and Weinberg, R.J. (2012). Ultrastructure of synapses in the mammalian brain. Cold Spring Harb. Perspect. Biol. *4*, a005587.

Heckscher, E.S., Lockery, S.R., and Doe, C.Q. (2012). Characterization of Drosophila larval crawling at the level of organism, segment, and somatic body wall musculature. J. Neurosci. *32*, 12460–12471.

Heckscher, E.S., Zarin, A.A., Faumont, S., Clark, M.Q., Manning, L., Fushiki, A., Schneider-Mizell, C.M., Fetter, R.D., Truman, J.W., Zwart, M.F., et al. (2015). Even-skipped(+) interneurons are core components of a sensorimotor circuit that maintains left-right symmetric muscle contraction amplitude. Neuron *88*, 314–329.

Hinckley, C.A., Alaynick, W.A., Gallarda, B.W., Hayashi, M., Hilde, K.L., Driscoll, S.P., Dekker, J.D., Tucker, H.O., Sharpee, T.O., and Pfaff, S.L. (2015). Spinal locomotor circuits develop using hierarchical rules based on motorneuron position and identity. Neuron *87*, 1008–1021.

Johnson, B.R., Schneider, L.R., Nadim, F., and Harris-Warrick, R.M. (2005). Dopamine modulation of phasing of activity in a rhythmic motor network: contribution of synaptic and intrinsic modulatory actions. J. Neurophysiol. *94*, 3101–3111.

Kiehn, O. (2011). Development and functional organization of spinal locomotor circuits. Curr. Opin. Neurobiol. *21*, 100–109.

Klapoetke, N.C., Murata, Y., Kim, S.S., Pulver, S.R., Birdsey-Benson, A., Cho, Y.K., Morimoto, T.K., Chuong, A.S., Carpenter, E.J., Tian, Z., et al. (2014). Independent optical excitation of distinct neural populations. Nat. Methods *11*, 338–346.

Lacin, H., and Truman, J.W. (2016). Lineage mapping identifies molecular and architectural similarities between the larval and adult Drosophila central nervous system. eLife *5*, e13399.

Laine, C.M., Martinez-Valdes, E., Falla, D., Mayer, F., and Farina, D. (2015). Motor neuron pools of synergistic thigh muscles share most of their synaptic input. J. Neurosci. *35*, 12207–12216.

Landgraf, M., Bossing, T., Technau, G.M., and Bate, M. (1997). The origin, location, and projections of the embryonic abdominal motorneurons of Drosophila. J. Neurosci. *17*, 9642–9655.

Landgraf, M., Jeffrey, V., Fujioka, M., Jaynes, J.B., and Bate, M. (2003). Embryonic origins of a motor system: motor dendrites form a myotopic map in Drosophila. PLoS Biol. *1*, E41.

Li, H.-H., Kroll, J.R., Lennox, S.M., Ogundeyi, O., Jeter, J., Depasquale, G., and Truman, J.W. (2014). A GAL4 driver resource for developmental and behavioral studies on the larval CNS of Drosophila. Cell Rep. *8*, 897–908.

Liu, W.W., and Wilson, R.I. (2013). Glutamate is an inhibitory neurotransmitter in the Drosophila olfactory system. Proc. Natl. Acad. Sci. USA *110*, 10294–10299.

Luan, H., Peabody, N.C., Vinson, C.R., and White, B.H. (2006). Refined spatial manipulation of neuronal function by combinatorial restriction of transgene expression. Neuron *52*, 425–436.

Machado, T.A., Pnevmatikakis, E., Paninski, L., Jessell, T.M., and Miri, A. (2015). Primacy of flexor locomotor pattern revealed by ancestral reversion of motor neuron identity. Cell *162*, 338–350.

Marley, R., and Baines, R.A. (2011). Whole-cell patch recording from *Drosophila* larval neurons. Cold Spring Harb. Protoc. *2011*, 1124–1127.

Matsushima, T., Tegnér, J., Hill, R.H., and Grillner, S. (1993). GABAB receptor activation causes a depression of low- and high-voltage-activated Ca2+ currents, postinhibitory rebound, and postspike afterhyperpolarization in lamprey neurons. J. Neurophysiol. *70*, 2606–2619.

Mauss, A.S., Meier, M., Serbe, E., and Borst, A. (2014). Optogenetic and pharmacologic dissection of feedforward inhibition in Drosophila motion vision. J. Neurosci. *34*, 2254–2263.

McLean, D.L., and Dougherty, K.J. (2015). Peeling back the layers of locomotor control in the spinal cord. Curr. Opin. Neurobiol. *33*, 63–70.

McLean, D.L., Fan, J., Higashijima, S., Hale, M.E., and Fetcho, J.R. (2007). A topographic map of recruitment in spinal cord. Nature *446*, 71–75.

McLean, D.L., Masino, M.A., Koh, I.Y.Y., Lindquist, W.B., and Fetcho, J.R. (2008). Continuous shifts in the active set of spinal interneurons during changes in locomotor speed. Nat. Neurosci. *11*, 1419–1429.

Miri, A., Azim, E., and Jessell, T.M. (2013). Edging toward entelechy in motor control. Neuron *80*, 827–834.

Nern, A., Pfeiffer, B.D., and Rubin, G.M. (2015). Optimized tools for multicolor stochastic labeling reveal diverse stereotyped cell arrangements in the fly visual system. Proc. Natl. Acad. Sci. USA *112*, E2967–E2976.

Ohyama, T., Schneider-Mizell, C.M., Fetter, R.D., Aleman, J.V., Franconville, R., Rivera-Alba, M., Mensh, B.D., Branson, K.M., Simpson, J.H., Truman, J.W., et al. (2015). A multilevel multimodal circuit enhances action selection in Drosophila. Nature *520*, 633–639.

Okado, N., Homma, S., Ishihara, R., and Kohno, K. (1990). Distribution patterns of dendrites in motor neuron pools of lumbosacral spinal cord of the chicken. Anat. Embryol. (Berl.) *182*, 113–121.

Percival, D.B., and Walden, A.T. (1993). Spectral Analysis for Physical Applications (Cambridge University Press).

Pfeiffer, B.D., Ngo, T.-T.B., Hibbard, K.L., Murphy, C., Jenett, A., Truman, J.W., and Rubin, G.M. (2010). Refinement of tools for targeted gene expression in Drosophila. Genetics *186*, 735–755.

Pratt, C.A., Chanaud, C.M., and Loeb, G.E. (1991). Functionally complex muscles of the cat hindlimb. IV. Intramuscular distribution of movement command signals and cutaneous reflexes in broad, bifunctional thigh muscles. Exp. Brain Res. *85*, 281–299.

Pulver, S.R., and Griffith, L.C. (2010). Spike integration and cellular memory in a rhythmic network from Na+/K+ pump current dynamics. Nat. Neurosci. *13*, 53–59.

Pulver, S.R., Bayley, T.G., Taylor, A.L., Berni, J., Bate, M., and Hedwig, B. (2015). Imaging fictive locomotor patterns in larval Drosophila. J. Neurophysiol. *114*, 2564–2577.

Rohrbough, J., and Broadie, K. (2002). Electrophysiological analysis of synaptic transmission in central neurons of Drosophila larvae. J. Neurophysiol. *88*, 847–860.

Romanes, G.J. (1964). The motor pools of the spinal cord. Prog. Brain Res. *11*, 93–119.

Sato, M., Kojima, T., Michiue, T., and Saigo, K. (1999). Bar homeobox genes are latitudinal prepattern genes in the developing Drosophila notum whose expression is regulated by the concerted functions of decapentaplegic and wingless. Development *126*, 1457–1466.

Schaefer, J.E., Worrell, J.W., and Levine, R.B. (2010). Role of intrinsic properties in Drosophila motoneuron recruitment during fictive crawling. J. Neurophysiol. *104*, 1257–1266.

Schindelin, J., Arganda-Carreras, I., Frise, E., Kaynig, V., Longair, M., Pietzsch, T., Preibisch, S., Rueden, C., Saalfeld, S., Schmid, B., et al. (2012). Fiji: an open-source platform for biological-image analysis. Nat. Methods *9*, 676–682.

Stepien, A.E., Tripodi, M., and Arber, S. (2010). Monosynaptic rabies virus reveals premotor network organization and synaptic specificity of cholinergic partition cells. Neuron *68*, 456–472.

Sürmeli, G., Akay, T., Ippolito, G.C., Tucker, P.W., and Jessell, T.M. (2011). Patterns of spinal sensory-motor connectivity prescribed by a dorsoventral positional template. Cell *147*, 653–665.

Sweeney, S.T., Broadie, K., Keane, J., Niemann, H., and O'Kane, C.J. (1995). Targeted expression of tetanus toxin light chain in Drosophila specifically eliminates synaptic transmission and causes behavioral defects. Neuron *14*, 341–351.

Talpalar, A.E., Endo, T., Löw, P., Borgius, L., Hägglund, M., Dougherty, K.J., Ryge, J., Hnasko, T.S., and Kiehn, O. (2011). Identification of minimal neuronal networks involved in flexor-extensor alternation in the mammalian spinal cord. Neuron *71*, 1071–1084.

Talpalar, A.E., Bouvier, J., Borgius, L., Fortin, G., Pierani, A., and Kiehn, O. (2013). Dual-mode operation of neuronal networks involved in left-right alternation. Nature *500*, 85–88.

Taylor, A.L., Cottrell, G.W., Kleinfeld, D., and Kristan, W.B., Jr. (2003). Imaging reveals synaptic targets of a swim-terminating neuron in the leech CNS. J. Neurosci. *23*, 11402–11410.

Tripodi, M., Stepien, A.E., and Arber, S. (2011). Motor antagonism exposed by spatial segregation and timing of neurogenesis. Nature *479*, 61–66.

Wang, W.-C., and McLean, D.L. (2014). Selective responses to tonic descending commands by temporal summation in a spinal motor pool. Neuron *83*, 708–721.

Zwart, M.F., Randlett, O., Evers, J.F., and Landgraf, M. (2013). Dendritic growth gated by a steroid hormone receptor underlies increases in activity in the developing Drosophila locomotor system. Proc. Natl. Acad. Sci. USA *110*, E3878–E3887.

Neuron, Volume *91*

Supplemental Information

Selective Inhibition Mediates

the Sequential Recruitment of Motor Pools

Maarten F. Zwart, Stefan R. Pulver, James W. Truman, Akira Fushiki, Richard D. Fetter, Albert Cardona, and Matthias Landgraf

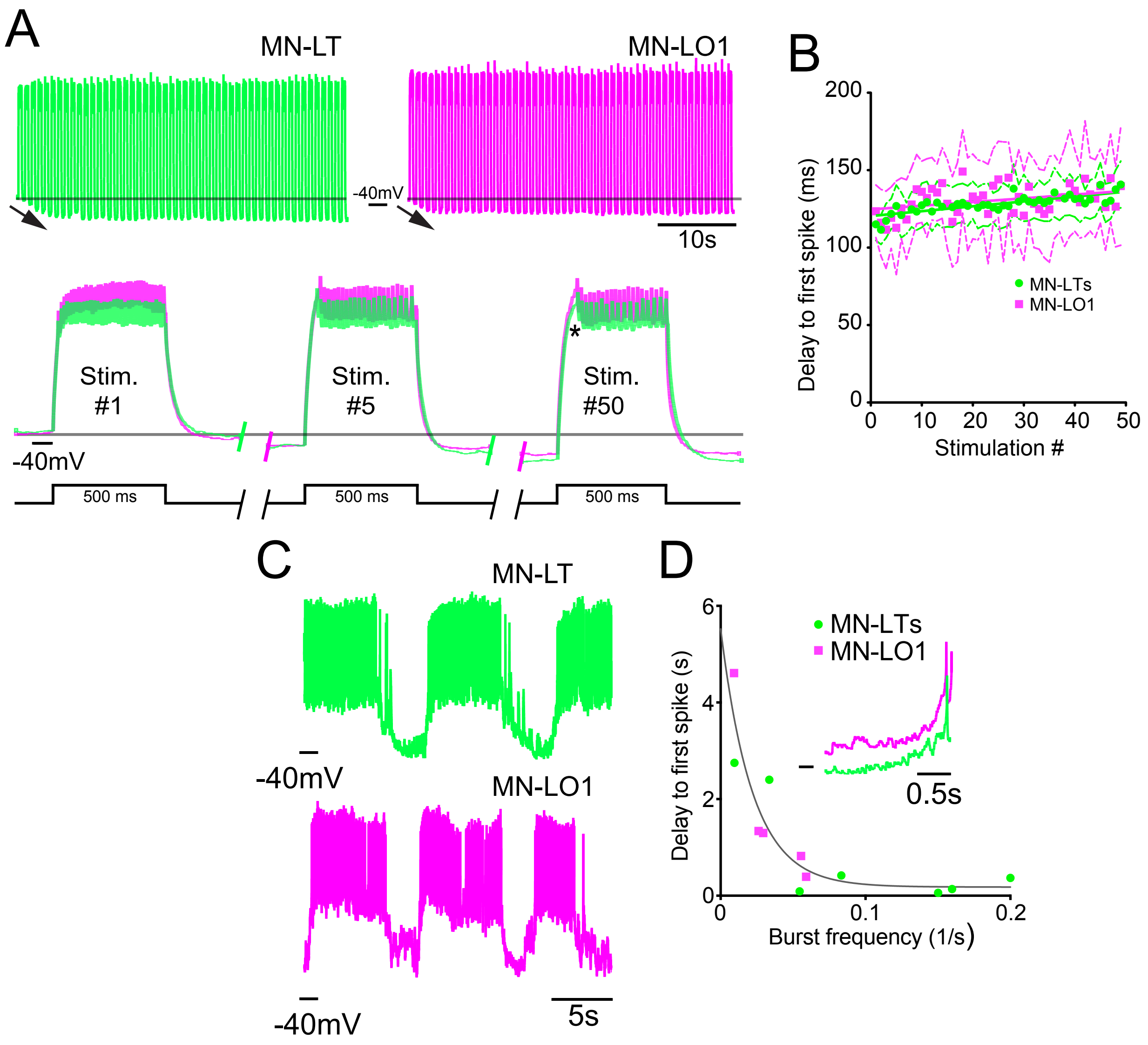

Figure S1, related to Figure 1. Motor neuron intrinsic properties do not contribute to the generation of the intrasegmental motor pattern underlying larval crawling.
(A) Top panels represent example traces of current clamp recordings of an MN-LT and MN-LO1 while repeatedly injecting 20 pA of current for 500 ms per stimulation. Horizontal line indicates resting membrane potential before experiment; arrow emphasizes downward trend after repeated stimulation. Bottom panels show overlaid traces of experiment for stimulation #1, #5 and #50. Asterisk in #50 indicates the change in the delay to first spike. (B) Quantification of delay to first spike as a function of stimulation number for MN-LT and MN-LO1. Plot shows mean delay to first spike (squares, circles) ± SEM (dashed lines). Solid lines indicate linear regression fits. There is no statistically significant difference between either the slopes (p=0.77) or intercepts (p=0.51) of the two fits. n=5 for MN-LO1, n=9 for MN-LTs. (C) Example traces of current clamp recordings of an MN-LT and MN-LO1 during fictive crawling in two different preparations. Motoneurons fire action potentials as the result of endogenous activity within the motor system. (D) Quantification of delay to first spike, as measured from the start of depolarization to the first action potential, as a function of burst frequency. Solid grey line indicates non-linear fit of the data. One curve fits both data sets best (p=0.17). Inset is expanded view of traces in (C) showing similar delay to first spike. n=5 for MN-LO1, n=7 for MNs-LT.

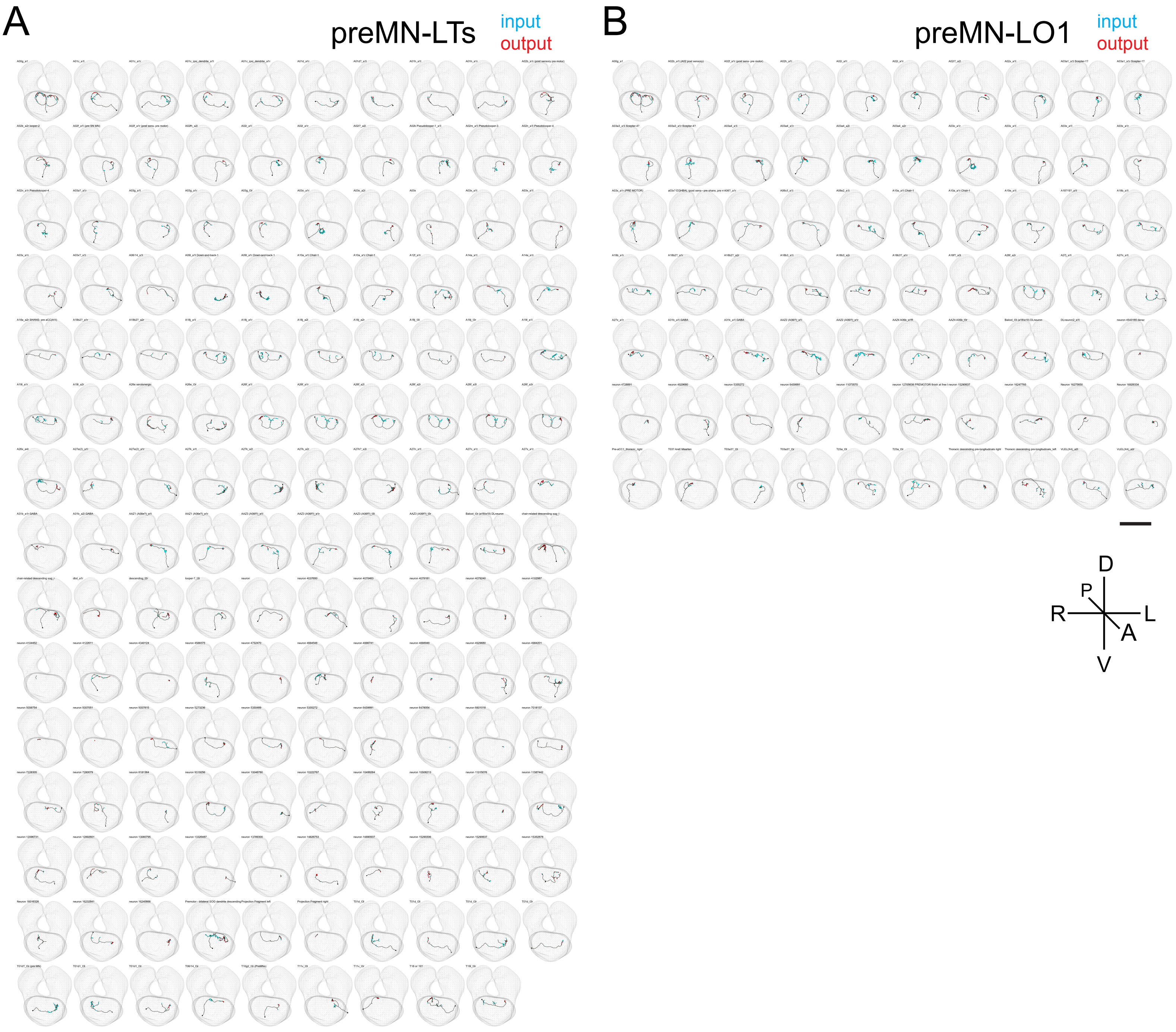

Figure S2, related to Figure 2. Functionally distinct motor neurons receive divergent input.
(A, B) Anterior views of individual reconstructions of all premotor interneurons reconstructed for this study. Scale bar indicates 50 µm.

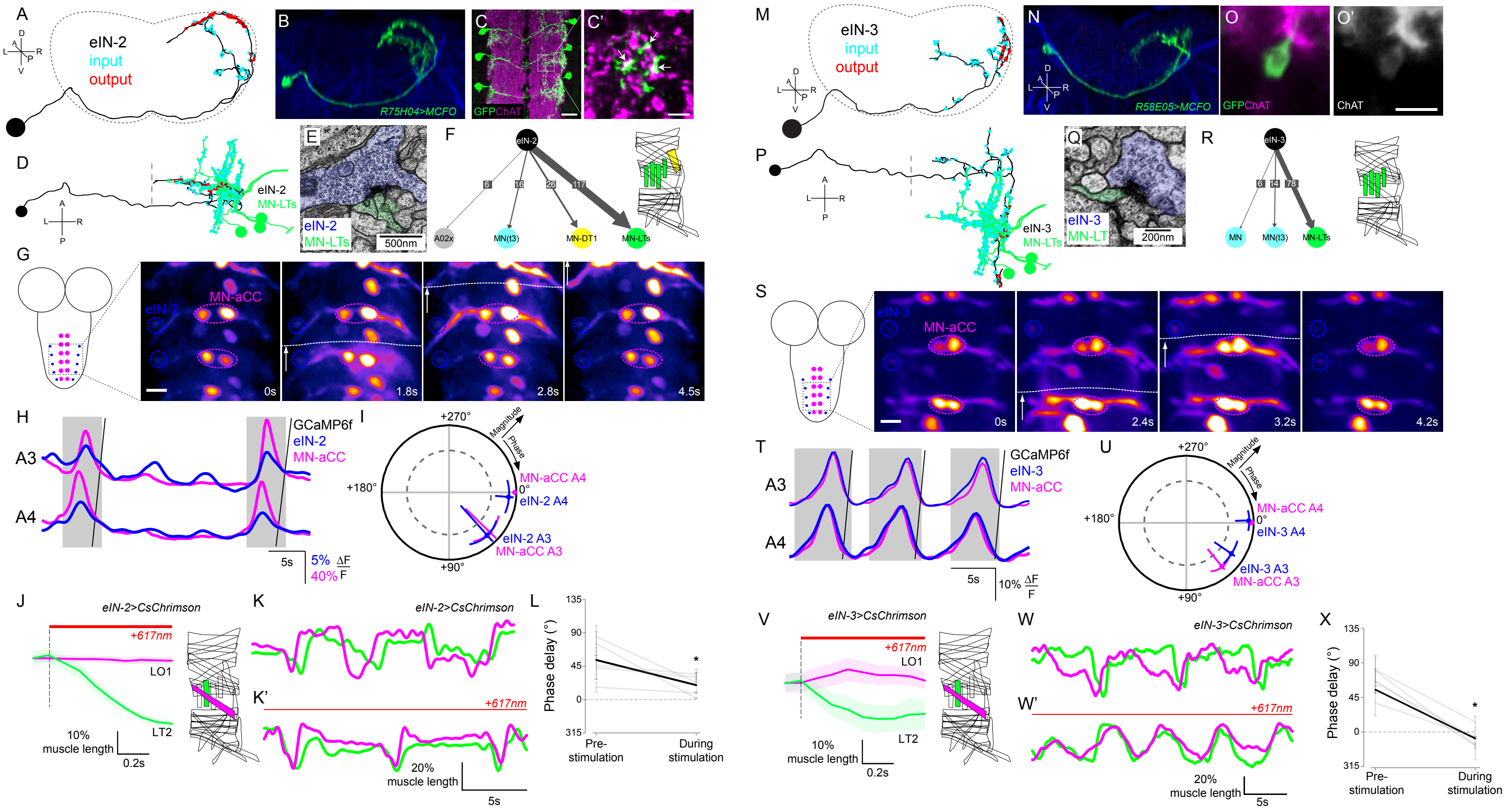

Figure S3, related to Figure 3. eIN-2 and eIN-3 innervate MN-LTs and are recruited during locomotion. Posterior view of EM reconstruction and light microscopy data of eIN-2 (A,B) and eIN-3 (M,N). (C, O) Immunohistochemical labeling of SS02065>myrGFP (C) and SS01379>GFP (O) showing pronounced ChAT staining. (D, P) Dorsal view of an eIN-2 (D) and eIN-3 (P) innervating contralateral MN-LT1 - MN-LT4. (E, Q) Electron micrograph showing the apposition of eIN-2 and two LT motoneurons (E) and an eIN-3 with a single MN-LT (Q), with synaptic vesicles and the presynaptic density clearly visible. (F, R) Connectivity diagram of eIN-2 and eIN-3. eIN-2 also innervates the DT1 motoneuron, which innervates a muscle of similar orientation as the MNs-LT. Blue motoneuron innervates unknown muscles (segmental identity in brackets), grey node indicates interneuron. Included in this diagram are all postsynaptic neurons with a connection to eIN-2 and eIN-3 of more than 5 synapses. Muscle diagram indicates identity of known target muscles. (G-I, S-U) eIN-2 and eIN-3 show wave-like activity during fictive crawling. (G, S) Stills showing GCaMP6f activity in eIN-2 and eIN-3 and aCC motoneurons as indicated in schematic, quantified in (H) and (T). (I, U) Coherency between eIN-2 and eIN-3 and aCC motoneurons in segments A3 and A4. (J, V) Acute high-intensity optogenetic stimulation (617 nm, $1.1 mW/mm^2$) of eIN-2 and eIN-3 induces contraction of the transverse muscles, as measured by muscle-imaging experiments in semi-intact preparations (see Experimental Procedures). LO1 does not contract in response to optogenetic stimulation of eIN-2 or eIN-3. (K, W) Low-level chronic stimulation of eIN-2 and eIN-3 (617 nm, $0.01 mW/mm^2$) causes the transverse muscles to contract earlier in the locomotor cycle. (K, W) Quantification of contraction of LO1 and LT2 muscles pre-stimulation, (K', W') during stimulation. (L, X) The phase delay between LO1 and LT2 is reduced in response to low-level chronic stimulation of eIN-2 (L) and eIN-3 (X). Grey lines indicate individual preparations, black line represents mean. Hotelling paired test, $p<0.05$ for (L) and (X). n=10 stimulations for (J) and (V), n=5 animals for all other experiments. Data are represented as mean ± 95% CI in (I) and (U), mean ± SD in (J), (L), (V), and (X). Scale bars in (C), (G), (O'), and (S) indicate 10µm, 1µm in (C').

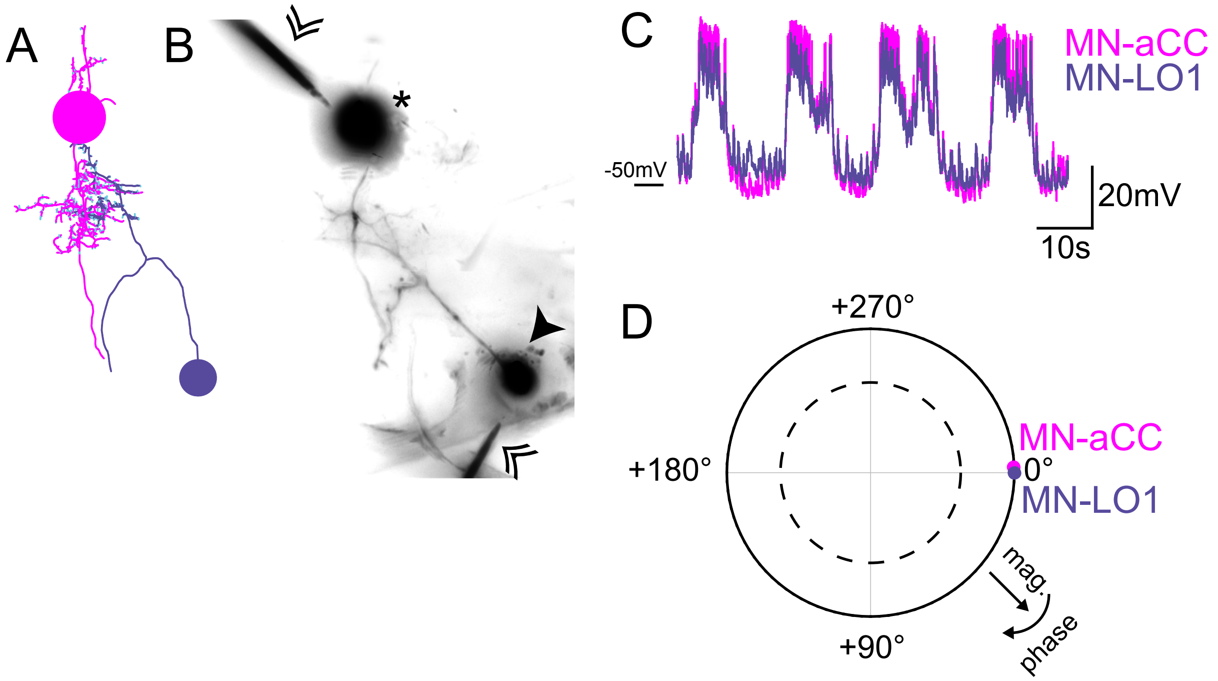

Figure S4, related to Figure 3. MN-LO1 is active in phase with MN-aCC.
(A) Dorsal view of EM reconstruction of MN-aCC (magenta) and MN-LO1 (violet) within the same segment. (B) Example recording of an MN-aCC (asterisk) and an MN-LO1 (arrowhead), showing recording electrodes (chevrons). Cells are visualized by Alexa 568 dye added to the intracellular solution. (C) Traces of simultaneous whole cell recording in current clamp of MN-aCC (magenta) and MN-LO1 (violet), showing membrane voltage fluctuations and action potentials that occur as the result of spontaneous fictive crawling. (D) Coherency between MN-aCC motoneuron and MN-LO1. MN-aCC and MN-LO1 are highly coherent with one another and are very close in phase. Data are represented as mean ± 95% CI, n=4.

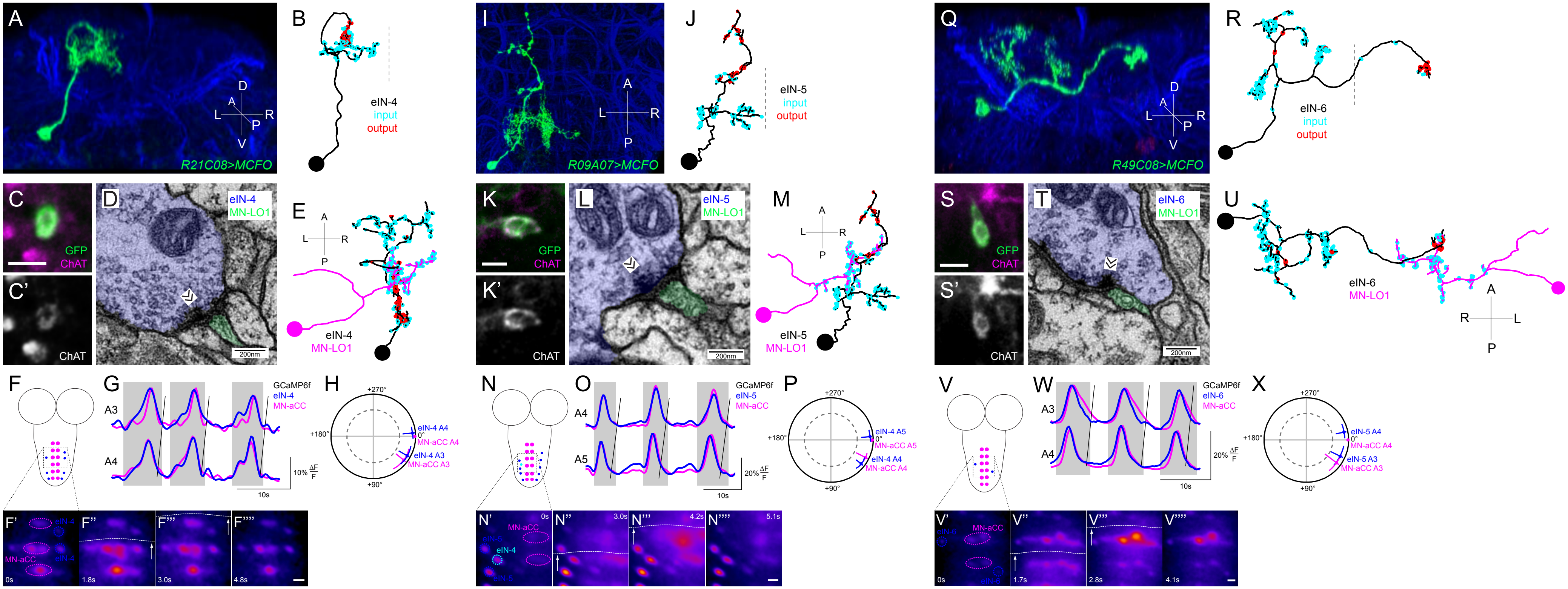

Figure S5, related to Figure 3. eIN-4, eIN-5 and eIN-6 innervate MN-LO1 and are recruited during locomotion.
Posterior (A, Q) and dorsal view (I) of light microscopy data and EM reconstructions (B, J, R) of eINs innervating MN-LO1. (C, K, S) Immunohistochemical labeling of SS01956>myrGFP (C), R09A07>myrGFP (K) and SS01404>myrGFP (S) showing pronounced ChAT staining. (D, L, T) Electron micrograph showing the apposition of eINs and MN-LO1, with synaptic vesicles and the presynaptic density clearly visible (chevrons). (E, M, U) Dorsal view of EM reconstructions of eINs innervating MN-LO1, either ipsilaterally (E, M) or contralaterally (U). (F-H, N-P, V-X) eINs show wave-like activity during fictive crawling. (F'-F'''', N'-N'''', V'-V''''). Stills showing GCaMP6f activity in eINs and aCC motoneurons as indicated in schematics (F, N, V), quantified in (G, O, W). (H, P, X) Coherency between eINs and aCC motor neurons in segment A3 and A4 or A4 and A5. Data are represented as mean ± 95% CI in (H), (P), and (X). n=5 for GCaMP imaging experiments, scale bars represent 10µm in (C, F''''), 5µm in (K, N'''', S, and V'''').

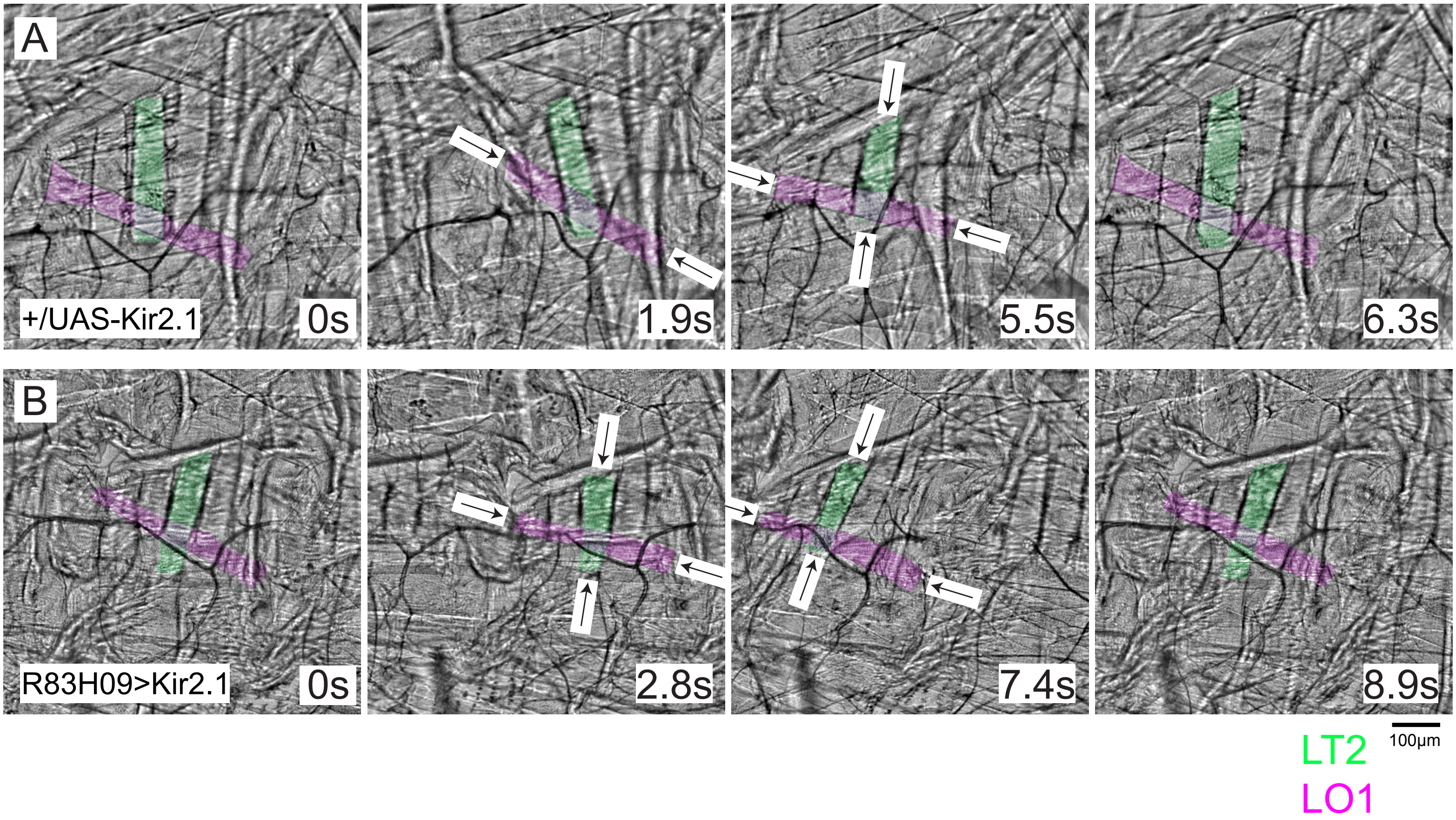

Figure S6, related to Figure 6. Inhibiting iIN-1 leads to a loss of the intrasegmental motor pattern. (A, B) Stills of imaging experiments to quantify muscle contraction patterns of LT2 (green) and LO1 (magenta) in a +/UAS-Kir2.1 control (A) and R83H09>Kir2.1 (B) preparation. Arrows indicate muscles contracting. See also Figure 6.
